# RTN4B‐mediated suppression of Sirtuin 2 activity ameliorates β‐amyloid pathology and cognitive impairment in Alzheimer's disease mouse model

**DOI:** 10.1111/acel.13194

**Published:** 2020-07-23

**Authors:** Yan Wang, Jing‐Qi Yang, Ting-Ting Hong, Yuan‐Hong Sun, Hai‐Li Huang, Feng Chen, Xiong‐Jin Chen, Hui‐Yi Chen, Shan‐Shan Dong, Li‐Li Cui, Tie‐Lin Yang

**Affiliations:** ^1^ Key Laboratory of Biomedical Information Engineering of Ministry of Education Biomedical Informatics & Genomics Center School of Life Science and Technology Xi'an Jiaotong University Xi'an China; ^2^ Guangdong Key Laboratory of Age‐Related Cardiac and Cerebral Diseases Affiliated Hospital of Guangdong Medical University Zhanjiang China; ^3^ Department of Pharmacology and Neuroscience University of North Texas Health Science Center Fort Worth TX USA; ^4^ Institute of Plastic Surgery Affiliated Hospital of Guangdong Medical University Zhanjiang China

**Keywords:** Alzheimer's disease, *APP*/*PS1* transgenic mice, Aβ, BACE1, RTN4B, Sirtuin 2

## Abstract

Sirtuin 2 (SIRT2) is an NAD+ dependent deacetylase that is the most abundant sirtuin protein in the brain. Accumulating evidence revealed the role of SIRT2 in a wide range of biological processes and age‐related diseases. However, the pivotal mechanism of SIRT2 played in Alzheimer's disease (AD) remains unknown. Here, we report that pharmacological inactivation of SIRT2 has a beneficial effect in AD. The deacetylase inhibitor of SIRT2 rescued the cognitive impairment in amyloid precursor protein/presenilin 1 transgenic mouse (*APP*/*PS1* mouse), and the BACE1 cleavage was weakened to reduce the β‐amyloid (Aβ) production in the hippocampus. Moreover, we firstly identified that Reticulon 4B (RTN4B) played a crucial role between SIRT2/BACE1 regulation in AD. RTN4B, as a deacetylation substrate for SIRT2, the deacetylation by SIRT2 drived the ubiquitination and degradation of RTN4B and then the disturbed RTN4B interacted with and influenced the expression of BACE1. When we overexpressed RTN4B in neurons of the hippocampus in the AD mouse model, the abnormal Aβ accumulation and cognitive impairment were ameliorated, consistent with the results of SIRT2 inhibition in vivo. Moreover, we showed that the regulatory effect of SIRT2 on BACE1 is dependent on RTN4B. When RTN4B was knocked down, the effects of SIRT2 inhibition on the BACE1 level, Aβ pathology, and AD‐liked behaviors were also blocked. Collectively, we provide evidence that SIRT2 may be a potential target for AD; the new found SIRT2/RTN4B/BACE1 pathological pathway is one of the critical mechanisms for the improvement of SIRT2 on AD.

## Graphical abstract

The repression of SIRT2 deacetylase activity could ameliorate Aβ pathology and cognitive deficits in the Alzheimer's Disease (AD) mouse model. As for the mechanism, SIRT2 influences the β‐secretase 1 (BACE1) by directly deacetylates reticulon 4B protein (RTN4B), thus affect the production of Aβ, finally contribute to the AD progress. These data marked that SIRT2/RTN4B/BACE1 is a new critical pathological pathway in recusing AD.

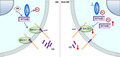

## 1 INTRODUCTION

Alzheimer's disease (AD) is the most common neurodegenerative disorder and the most prevalent cause of dementia, representing a worldwide epidemic problem in contemporary health care (World Health Organization, 2015). At present, AD could only take measures with symptomatic, lacking effective methods of early diagnosis and treatment; the underlying mechanisms of this disease remain incompletely defined (Alzheimer’s Association, 2019; Long & Holtzman, 2019). The primary neuropathological signs of AD, including the extracellular deposition of amyloid‐β (Aβ) peptides and intracellular neurofibrillary tau tangles, are closely associated with synapse and neuron loss, ultimately memory impairment in AD (De Strooper & Karran, 2016; Palop & Mucke, 2016; Zott, Busche, Sperling, & Konnerth, 2018). Although Aβ theory has been challenged due to the setback of clinical experiments with Aβ as the target, a great many convincing pieces of evidence support that Aβ is the most critical target of AD, and its pathogenesis in AD still acquired a greater depth of understanding (Rao, Asch, Carr, & Yamada, 2020).

The sirtuin family of proteins, which includes seven mammalian members, are famous protein deacetylases that participate in considerable biological and pathological processes, highlighting their crucial physiological functions (Finkel, Deng, & Mostoslavsky, 2009; Gomes, Leal, Mendes, Reis, & Cavadas, 2019; Lin et al., 2018). Among them, SIRT2 is the only sirtuin mainly located in the cytoplasm and abundantly expressed in the brain (Jayasena et al., 2016). Moreover, SIRT2 also accumulates in the aging central nervous system (CNS), marking its potential role in aging or related neurological diseases (Maxwell et al., 2011). Recently, a *SIRT2* polymorphism was associated with AD risk in different populations, providing evidence for the relationship between SIRT2 and AD from the perspective of genetics (Polito et al., 2013; Porcelli et al., 2013). Furthermore, *SIRT2* mRNA levels increased in the peripheral blood of patients with AD (Wongchitrat et al., 2019), and SIRT2 levels escalated alongside the decreased acetylation of its recognized substrate α‐tubulin in the AD brain (Silva, Esteves, Oliveira, & Cardoso, 2016). Moreover, recent studies showed that the interference of SIRT2 mitigated AD‐like recognition deficits in both the mouse model of neurodegenerative diseases and the aged‐accelerated mouse model (Biella et al., 2016; Diaz‐Perdigon et al., 2020). These above pieces of evidence suggested that SIRT2 might play a significant role in CNS and represent a potential drug target for AD. However, the molecular details underpinning the effects of SIRT2 in AD remain elusive.

In the present study, we aim to explore the potential effect of SIRT2 on the AD process and the hidden mechanism. We report that the repression of SIRT2 deacetylase activity ameliorates Aβ pathology and cognitive deficits in the AD mouse model. Furthermore, we provide detail mechanisms that SIRT2 deacetylates reticulon 4B protein (RTN4B) and then influences the β‐secretase 1 (BACE1) , ultimately contribute to the Aβ pathology. Our data demonstrate that targeting SIRT2 could be a rational strategy for AD, and RTN4B is the critical regulator of the SIRT2‐mediated Aβ metabolism for modifying the AD progression.

## 2 RESULTS

### 2.1 Inhibition of SIRT2 deacetylation activity has beneficial effects in the AD mouse model

**FIGURE 1:**
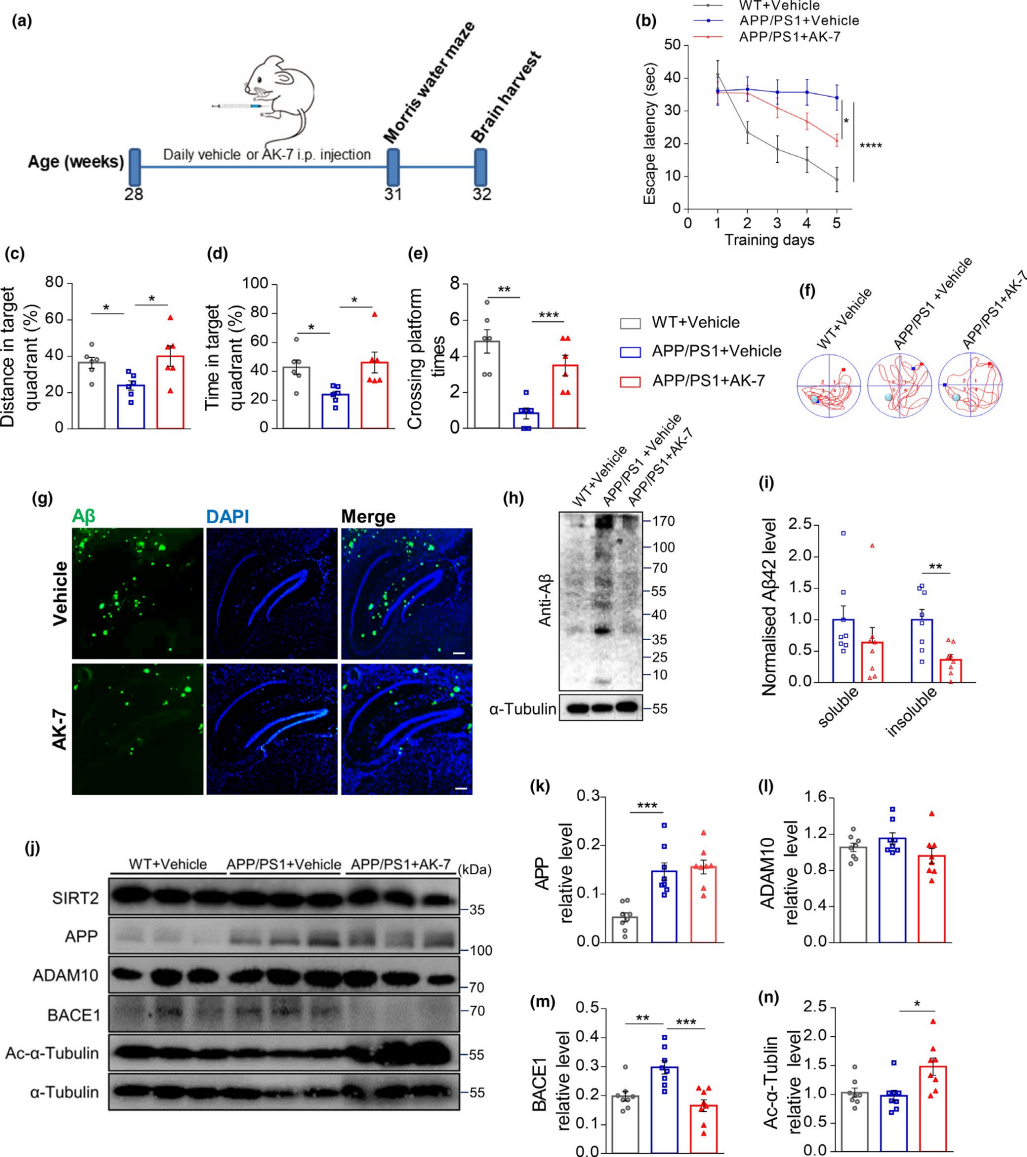
Inhibition of SIRT2 activity ameliorates AD‐associated pathology in *APP*/*PS1* mouse. (a) Illustration of timeline of the experimental flow. Seven‐month‐old *APP*/*PS* mice were treated with AK‐7 or vehicle, and age‐matched WT mice treated with vehicle were used as controls. (b) In the hidden platform test, the escape latency to find the platform was plotted against the days of training (*n* = 6 per group). (c–e) In the probe trial, the time, distance, and crossing times in the target quadrant where the platform was removed were recorded (*n* = 6 per group). (f) Representative swim trajectory of mice in the MWM test, the green circle indicates the hidden platform. The red dot indicates the start site, and the blue indicates the stop site. (g) Representative image of Aβ staining in the brain of an *APP*/*PS1* mouse treated with AK‐7 or vehicle (scale bar, 250 μm). (h) Representative western blot images of Aβ in the brain of an *APP*/*PS1* mouse treated with AK‐7 or vehicle. (i) ELISA was used to measure soluble and insoluble Aβ42 levels in the *APP*/*PS1* mouse brain (*n* = 8 per group). (j) Representative western blot images of SIRT2, APP, ADAM10, BACE1, acetylated‐α‐tubulin, and α‐tubulin. (k–n) Relative expression of the above proteins was analyzed (*n* = 8 per group). Data are presented as mean ± standard error of the mean (*SEM*). *p < 0.05, ** p < 0.01, *** p < 0.001, **** p < 0.0001. Non‐paired Student's *t* test for (i); one‐way ANOVA with Tukey's post hoc test for (c–e, k–n); two‐way ANOVA for (b)

To confirm whether interfering with the function of SIRT2 deacetylation could slow down the progression of AD‐like changes and behavior, we inhibited SIRT2 function using the selective, brain‐permeable inhibitor AK‐7. Our in vitro results first confirmed that as the increased AK‐7 concentration, the acetylation level of α‐tubulin also increased (Appendix S1, Figure S1). Next, to determine whether inhibition of SIRT2 may improve AD‐related behavioral deficits, the Morris water maze (MWM) test was carried out in the *APP*/*PS1* mouse. Our results showed that the 3‐week administration of AK‐7 (100 mg/kg, twice/d, intraperitoneally [i.p.,]) ameliorated the cognitive functional defect in 7‐month‐old *APP*/*PS1* mice compared to vehicle‐treated mice (Figure 1a–f). Meanwhile, the bodyweight not changed with the AK‐7 administration (Appendix S1, Figure S2).

To further investigate how SIRT2 influenced the cognitive behavior and its mechanism of AD, we assessed the hippocampal Aβ deposition of *APP*/*PS1* mouse with the effect of SIRT2 inactivation. As shown in Figure 1g,h, the SIRT2 inhibition reduced the Aβ load in the hippocampus of *APP*/*PS1* mice and reduced the aggregated form of Aβ. Moreover, repression of SIRT2 significantly reduced the insoluble form of Aβ42 (Figure 1i). These results above suggest that SIRT2 inhibition reduces Aβ42 abundance and aggregation. To further explore the mechanism underlying SIRT2 inhibition on Aβ pathology, we assayed the key secretases involved in APP processing. The results showed that SIRT2 inhibition reduced the expression of BACE1, which increased in *APP*/*PS1* mice compared to WT mice. Meanwhile, no significant change in APP and A disintegrin and metalloproteinase domain‐containing protein 10 (ADAM10) abundance was observed (Figure 1j–m). The activity inhibition effect of AK‐7 in vivo was confirmed by the increased acetylation level of α‐tubulin and unchanged SIRT2 expression (Figure 1n, Appendix S1, Figure S3). We further generated *SIRT2* knockout mice and found that depletion of *SIRT2* significantly reduced BACE1 levels in both the hippocampus and cortex in 15‐month‐old mice (Appendix S1, Figure S4). Collectively, these results suggest that the inhibition of SIRT2 deacetylation activity may be effective against the cognitive impairment in AD mice, via a reduction of Aβ production by influencing BACE1.

### 2.2 Characterization of SIRT2 interactions in vitro

**FIGURE 2:**
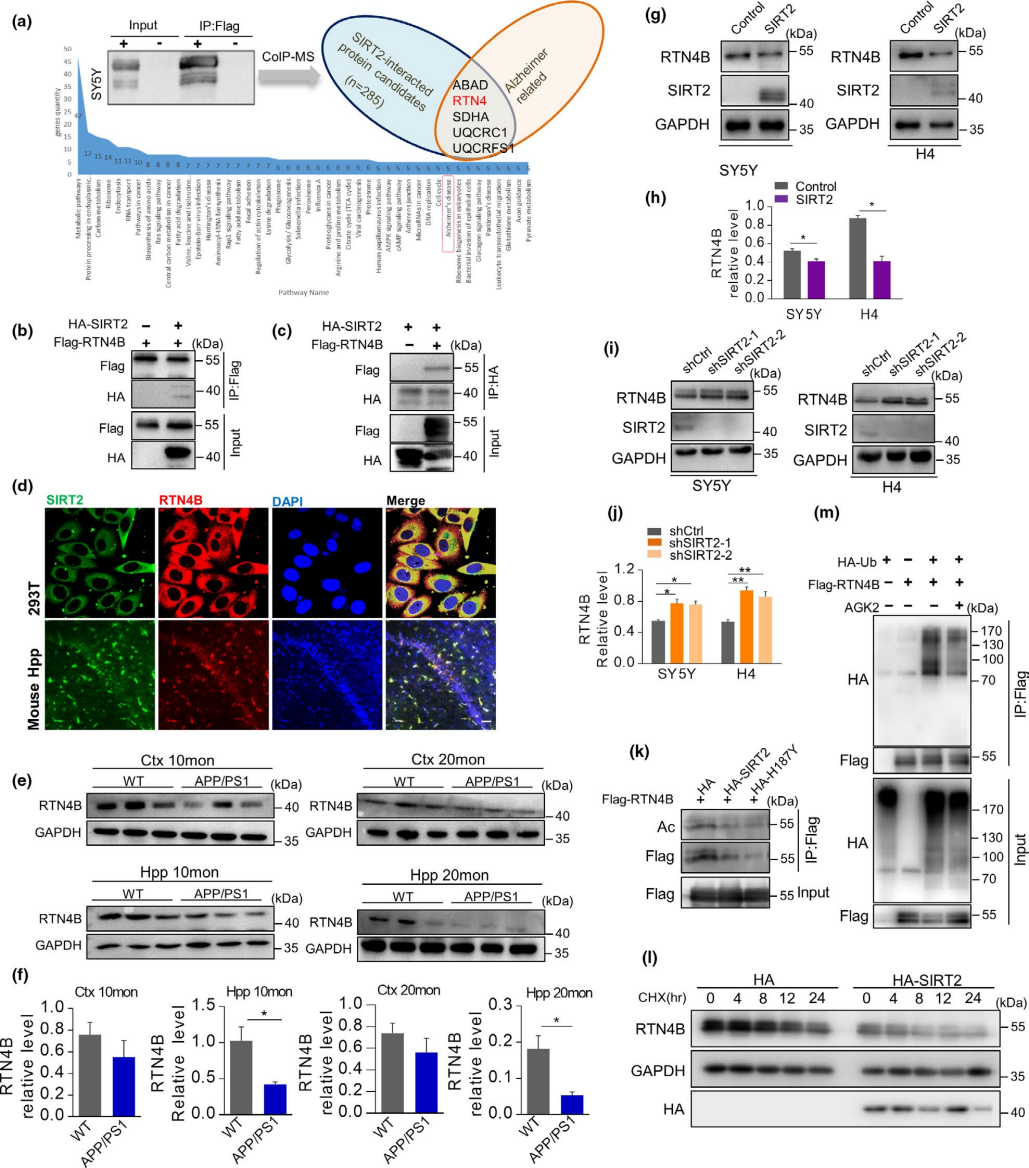
SIRT2 interacted with RTN4B and regulated its level through deacetylase activity. (a) Experimental flow chart of RTN4B discovery by mass spectrometry. (b–c) HA‐SIRT2 and Flag‐RTN4B plasmids were transiently transfected into 293T cells. Total proteins were IPed with HA or Flag antibodies and subsequently by western blot with HA or Flag antibodies. (d) Endogenous SIRT2 and RTN4B were stained using SIRT2 and RTN4B antibodies in 293T cells and hippocampal tissue from WT mouse brain (scale bar, 50 μm). (e and f) Representative western blot images and relative expression of RTN4B in the hippocampus (Hpp) and cortex (Ctr) from *APP*/*PS1* and WT mice at 10 and 20 months old (*n* = 3 per group). (g, h) SY5Y and H4 cells were transiently transfected with HA‐SIRT2 plasmids, and the RTN4B expression was detected by western blot. (i, j) SY5Y and H4 cells were infected with lentivirus‐mediated shSIRT2. RTN4B levels were determined by western blot. (k) Flag‐RTN4B and HA‐SIRT2 or SIRT2H187Y were transiently transfected into 293T cells, in the presence of a non‐sirtuin HDAC (histone deacetylase) inhibitor TSA. Total proteins were IPed with Flag and subsequently western blot with Ac and Flag antibodies. (l) HA or HA‐SIRT2 were transiently transfected into 293T cells, and cells were then treated with CHX at the indicated time points. RTN4B levels were determined by western blot analysis. (m) HA‐Ub and Flag‐RTN4B were transiently transfected into 293T cells. Total proteins were IPed with Flag and subsequently by western blot using HA and Flag antibodies. Data are presented as mean ± *SEM*. *p < 0.05, **p < 0.01. Non‐paired Student's *t* test for (f, h); one‐way ANOVA with Tukey's post hoc test for (j). Data in (b‐d, g‐m) are representative of 3–4 independent experiments

To identify how SIRT2 regulated BACE1 in AD progress, we performed co‐immunoprecipitation (Co‐IP) in the SIRT2‐overexpressing SY5Y cell line and conducted mass spectrometry (MS) analysis of the SIRT2 trapped proteins to screening the potential substrate of SIRT2 in the neural cell line. This proteomic approach finally identified 285 candidate SIRT2‐binding partners and some of which have been previously reported to interact with SIRT2 (Lin et al., 2013; Wang et al., 2014). We further focused on identifying the protein candidates with the Alzheimer's disease‐related and finally identified five proteins in the KEGG pathway analysis. Among them, RTN4 was identified that both have a direct relationship with BACE1 and act as the potential substrate of SIRT2 according to the results of informatics analysis (Figure 2a, Appendix S1, Figure S5, Table S1).

### 2.3 SIRT2 interacts with and regulates RTN4B

Considering RTN4 has three isoforms, we firstly evaluated which RTN4 isoform is the major targets of SIRT2, and the Co‐IP results showed that only RNT4B has the significant interaction with SIRT2 (Appendix S1, Figure S7). Then, reciprocal Co‐IP was further performed to confirm the interaction between them and the results showed that SIRT2 Co‐IPed with RTN4B, and vice versa, indicating a direct interaction with each other (Figure 2b,c). Moreover, SIRT2 and RTN4B also showed co‐location in both 293T cells and the hippocampus of WT mice (Figure 2d). Next, we also determined whether the protein level of RTN4B changed in AD. Our informatics analysis results show that RTN4 was reduced in the brain of AD patients (Appendix S1, Figure S6), and our results showed that the protein level of RTN4B also significantly reduced in *APP*/*PS1* mice compared to the WT control mice group, especially in the hippocampus of both 10‐month‐old and 20‐month‐old mice (Figure 2e,f).

Next, we investigated the regulatory relationship between SIRT2 and RTN4B. Two overexpressed and knockdown SIRT2 cell lines were established in SH‐SY5Y and H4 cells. The efficacy of the transfections was determined by visualization of green fluorescent protein (GFP) (Appendix S1, Figure S8). The results showed that overexpression of SIRT2 reduced the abundance of RTN4B in the two neural cell lines (Figure 2g,h), and when the level of SIRT2 decreased, RTN4B has up‐regulated accordingly (Figure 2i,j). This negative association was further supported in vivo that we observed the increased RTN4B expression in the hippocampus and cortex of the *SIRT2* knockout mice compared to the WT mice (Appendix S1, Figure S9). Moreover, we further evaluated the possible association between the protein expression of SIRT2 and RTN4 in the brain of AD patients based on the published data and found that the change direction of RTN4 and SIRT2 is opposite in some brain regions (Appendix S1, Figure S6). Collectively, these results suggest that SIRT2 can interact with and negatively regulate the protein level of RTN4B.

### 2.4 SIRT2 influences the ubiquitination and degradation of RTN4B by deacetylation

Since SIRT2 is a classical cytoplasmic deacetylase, and RTN4B has also been identified as a protein that has lysine acetylation site in a sizeable proteomic study (Choudhary et al., 2009). So we further evaluate whether SIRT2 regulated the RTN4B expression by deacetylation activity. We transfected 293T cells with Flag‐RTN4B and HA‐SIRT2 WT or deacetylation‐null mutants HA‐SIRT2 H187Y, and found that the acetylation statue of RTN4B can be decreased by SIRT2 but not the H187Y mutation of SIRT2 in the presence a non‐sirtuin HDAC inhibitor TSA (Figure 2k). These results indicate that RTN4B is a legitimate deacetylation substrate of SIRT2.

To further determine whether SIRT2 regulates the expression of RTN4B by post‐translational level, cycloheximide (CHX, 100 μg/ml) was used to prevent new protein synthesis, and the results show that the RTN4B expression decreased when cells were treated with CHX. Moreover, this decrease tended to be more noticeable when SIRT2 overexpressed suggested that SIRT2 plays a post‐translational regulatory role in RTN4B (Figure 2l). Acetylation and ubiquitination are commonly competing for post‐translational modifications that target the lysine residues of proteins. Then, we evaluated whether the acetylation of RTN4B could influence its ubiquitination, therefore influence its degradation as well. To prove this hypothesis, we immunoprecipitated RTN4B in 293T cells co‐transfected with HA‐Ub and Flag‐RTN4B, and the result showed that RTN4B can be ubiquitinated and that the inhibition of SIRT2 by AGK2 (10 μM, 12 hr) reduced the ubiquitination of RTN4B (Figure 2m).

### 2.5 SIRT2 regulates BACE1 via RTN4B in vitro

**FIGURE 3:**
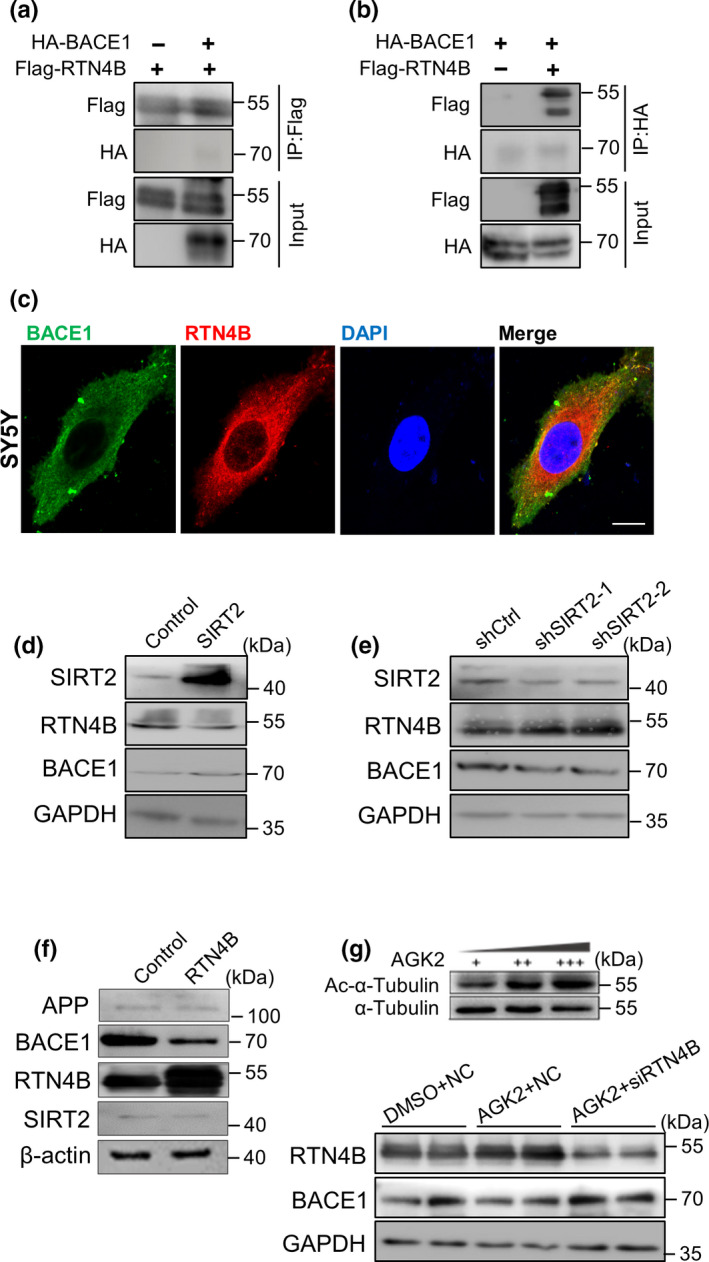
SIRT2 regulates the RTN4B/BACE1 axis in vitro. (a, b) HA‐BACE1 and Flag‐RTN4B plasmids were transiently transfected into 293T cells. Total proteins were IPed with HA and Flag antibodies and subsequently by western blot analysis using HA and Flag antibodies. (c) Endogenous BACE1 and RTN4B were stained using BACE1 and RTN4B antibodies in SY5Y cells (scale bar, 5 μm). (d, e) HA‐SIRT2 or shSIRT2 were transiently transfected into SY5Y cells, and RTN4B and BACE1 protein levels were determined by western blot. (f) Flag‐RTN4B plasmids were transiently transfected into 293T cells, and BACE1 protein levels were determined by western blot. (g) siRTN4B or negative control was transfected into SY5Y cells, then treat cells with AGK2 or DMSO, and RTN4B and BACE1 protein levels were determined by western blot. All panels are representative of 3–4 independent experiments

Some common domains of the RTN family have direct interaction with BACE1 (He et al., 2004), which prompted us to consider whether SIRT2 inhibition reduces BACE1 levels by increasing RTN4B expression. First, we confirmed that BACE1 interacts with RTN4B in 293T cells by reciprocal Co‐IP (Figure 3a,b). Subsequently, the assay of fluorescence colocalization in SY5Y cells shows that BACE1 and RTN4B colocalize mainly in the cytoplasm (Figure 3c). To determine whether SIRT2 regulates RTN4B and then influences the expression of BACE1, we overexpressed SIRT2 in SY5Y cells and evaluated RTN4B and BACE1 expression levels. As shown in Figure 3d, the increased SIRT2 expression can reduce RTN4B protein levels and increase BACE1 protein levels accordingly. Alternatively, the reduction of SIRT2 can increase RTN4B and decrease BACE1 levels (Figure 3e). We also assayed the mRNA level of *RTN4B* and *BACE1* and conformed that this regulation of SIRT2 only occurred at the protein level (Appendix S1, Figure S10). To ascertain whether overexpression of RTN4B could have a similar effect to SIRT2 inhibition, we overexpressed RTN4B in SY5Y cells and then examined BACE1 levels. The results showed that RTN4B overexpression reduced BACE1 levels (Figure 3f). We subsequently examined whether SIRT2 regulation of BACE1 is RTN4B‐dependent, *RTN4B* was first knocked down by small interfering RNA (siRNA) in SY5Y cell lines, and then the effect of SIRT2 inhibition by AGK2 (10 μM, 12 hr) on BACE1 abundance was assessed. The results showed that the influence of SIRT2 inhibition on BACE1 was abrogated on the premise of RTN4B reduction in SY5Y cells (Figure 3g). In brief, the above results suggest that SIRT2/RTN4B/BACE1 is a potential pathway by which SIRT2 could alleviate AD‐like pathology.

### 2.6 Overexpressed RTN4B in hippocampal neurons alleviates cognitive impairments in the AD mouse model

**FIGURE 4:**
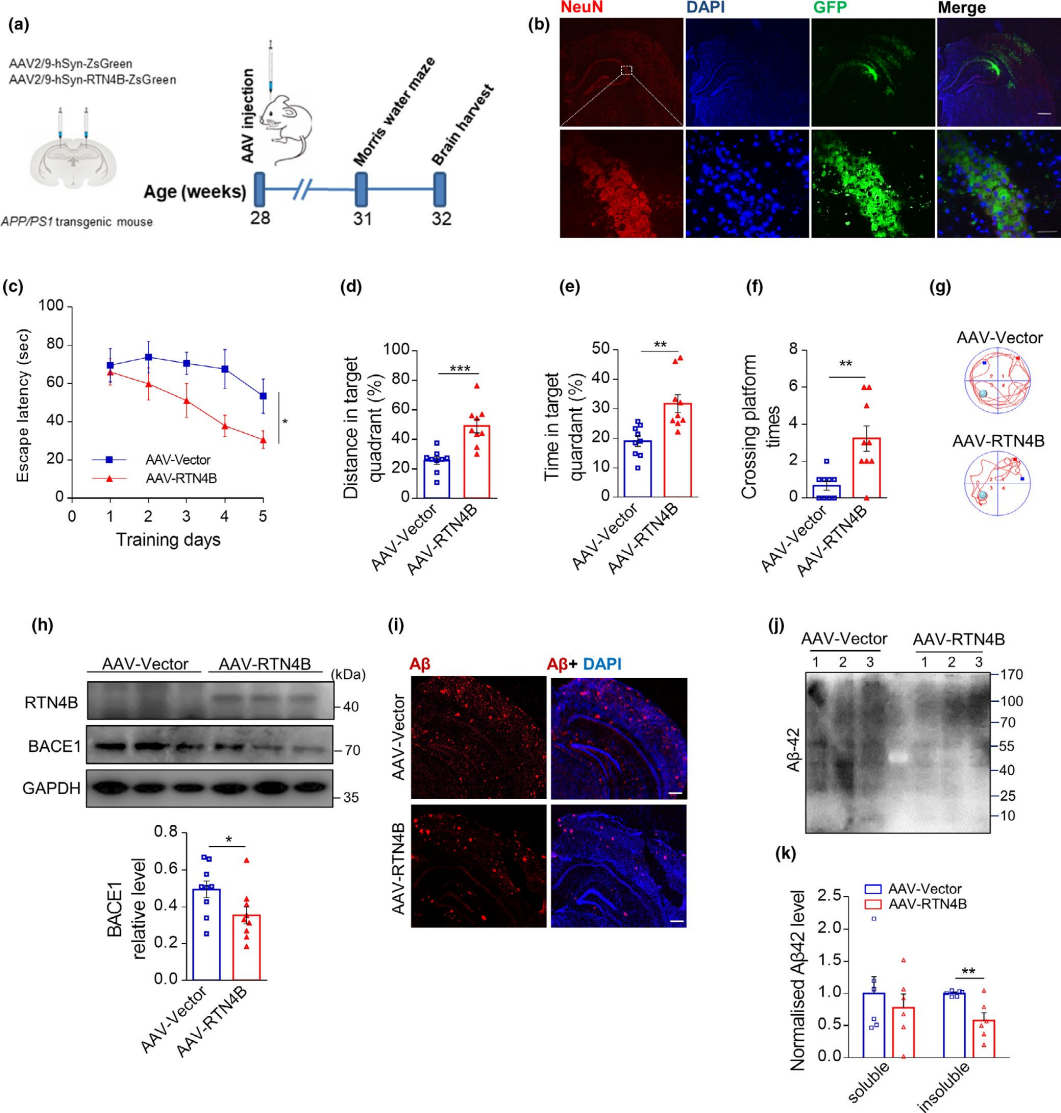
Overexpression of RTN4B rescued AD‐associated pathology in *APP*/*PS1* mice. (a) Illustration of bilateral stereotactic injection of AAV and timeline of the experimental flow. Seven‐month‐old *APP*/*PS* mice were injected with AAV in hippocampus. (b) The effect of the injection was assessed by immunofluorescence staining for NeuN cells (scale bar, upper 500 μm lower 50 μm). (c) In the hidden platform test, the escape latency to find the platform was plotted against the days of training (*n* = 9 per group). (d–f) In the probe trial, time, distance, and crossing times in the target quadrant where the platform was removed were recorded (*n* = 9 per group). (g) Representative swim trajectory of mice in the MWM test. The green circle indicates the hidden platform. The red dot indicates the start site, and the blue indicates the stop site. (h) Representative western blot images of RTN4B and BACE1 and relative expression of BACE1 (*n* = 9 per group). (i, j) Representative images of immunofluorescence staining and western blot of hippocampal Aβ (scale bar, 250 μm). (k) ELISA was used to measure soluble and insoluble Aβ42 levels in hippocampal tissue (*n* = 6 per group). Data are presented as mean ± *SEM*. *p < 0.05, ** p < 0.01, *** p < 0.001. Non‐paired Student's *t* test for (d–f, h, k); two‐way ANOVA for (b)

Next, to directly investigate the contribution of RTN4B to the AD‐like pathology in AD, we overexpressed RTN4B in the hippocampal neurons of *APP*/*PS1* mice by bilateral injection of an AAV2/9 virus equipped with a ZsGreen tag driven by neuron‐specific promoters (Figure 4a). First, we verified the effect of the virus using immunofluorescence analysis of brain sections 3 weeks post‐injection. As shown in Figure 4b, green fluorescence was co‐located with NeuN, suggesting that hippocampal neurons specifically expressed the virus. To evaluate the influence of RTN4B on hippocampal‐dependent learning and memory, we conducted the MWM test after 3 weeks of administration. The results show that the overexpressing RTN4B *APP*/*PS1* mice took less time to find the platform in the training trial. In the probe trial, RTN4B overexpressed groups increased the time and the distance in the target quadrant and the times crossing the platform, respectively (Figure 4c–g). As expected, the hippocampal RTN4B expression increased after virus injection compared to the control vector injection, and the protein level of BACE1 was suppressed in the hippocampus under the RTN4B overexpression (Figure 4h). To further assess the progression of Aβ pathology, we performed immunofluorescence and western blot analyses in the hippocampus, and the results showed that RTN4B overexpression reduced the number of Aβ plaques and aggregation in *APP*/*PS1* mice (Figure 4i,j). In parallel with the immunofluorescence result, the insoluble Aβ42 was also reduced by RTN4B overexpression using ELISA assay (Figure 4k). These results suggest that RTN4B overexpression rescued the cognitive impairments in *APP*/*PS1* mouse, suppressed BACE1 protein level, and ultimately reduced the production of Aβ.

### 2.7 The beneficial effects of SIRT2 inhibition in the AD mouse model are RTN4B dependent

**FIGURE 5:**
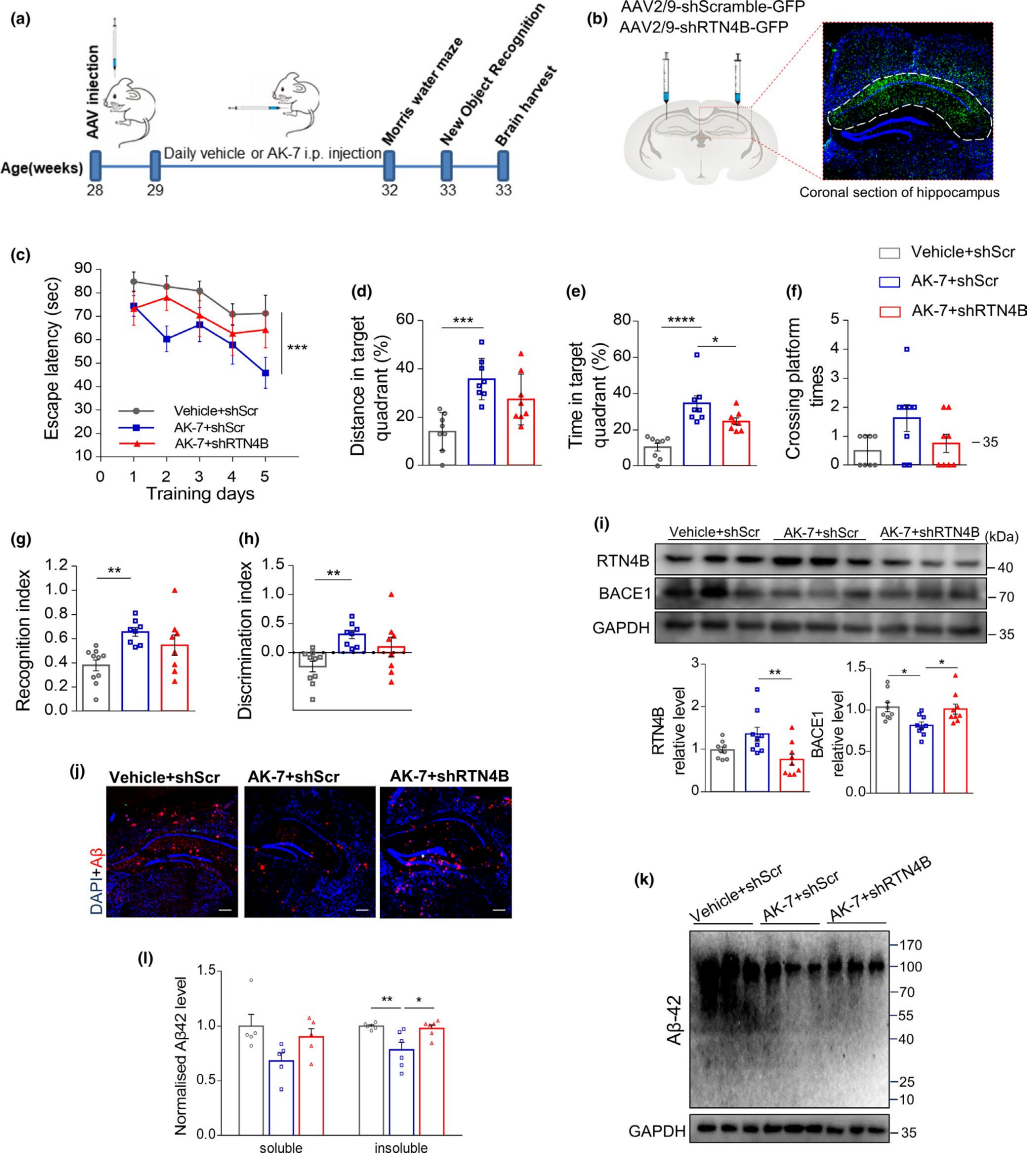
RTN4B‐dependent SIRT2 inhibition ameliorates AD‐associated pathology and improves cognition. (a) Illustration of the experimental timeline. 7‐month‐old *APP*/*PS1* mice were used. (b) Illustration of bilateral stereotactic injection of AAV and the efficiency of the viral stereotactic injection was assessed by immunofluorescence. (c) In the hidden platform test, the escape latency to find the platform was plotted against the days of training (*n* = 8 per group). (d–f) In the probe trial, time, distance, and crossing times in the target quadrant where the platform removed were recorded (*n* = 8 per group). (g, h) In the object recognition test, the recognition index and the discrimination index were recorded (*n* = 8‐10 per group). (i) Representative western blot images of RTN4B and BACE1 and Relative expression of the above proteins (*n* = 9 per group). (j, k) Representative images of immunofluorescence staining and western blot of Aβ in the hippocampus (scale bar, 250 μm). (l) ELISA was used to measure soluble and insoluble Aβ42 levels in hippocampal tissue (*n* = 5‐6 per group). Data are presented as mean ± *SEM*. *p < 0.05, ** p < 0.01, *** p < 0.001. One‐way ANOVA with Tukey's post hoc test for (d–h, i, l); two‐way ANOVA for (c)

From the in vitro and in vivo experiments above, we concluded that inhibition of SIRT2 could reduce BACE1 by increasing RTN4B. To further determine whether the beneficial effect of SIRT2 inhibition in the AD mouse model is RTN4B‐dependent, we knocked down the RTN4B and then examined whether the effect of SIRT2 inhibition is still effective in vivo (Figure 5a). The stereotactic injection of an AAV2/9 virus that contained shRTN4B sequence was conducted bilaterally in the *APP*/*PS1* mouse hippocampus to knockdown RTN4B, a virus containing a scrambled sequence was used as the control, and GFP was visualized to confirm the effect of the injection (Figure 5b). Then, MWM and novel object recognition test (NOR) were employed to verify the behavior cognition changes after intervention. As shown in Figure 5c–f, AK‐7 administration reduced the time spent finding the platform and increase the time and distance in the target quadrant. However, when RTN4B was knocked down, all of these improvements in behavior cognition were attenuated. With regard to the NOR test, a similar result was observed. AK‐7 administration increased the recognition index and discrimination index of *APP*/*PS1* mice compared with the placebo groups. However, the absence of RTN4B abrogated this behavior improvement (Figure 5g,h).

Furthermore, we analyzed the expression of RTN4B and BACE1 by western blot, and as expected, AK‐7 administration increased RTN4B and reduced BACE1. Nevertheless, when RTN4B was knocked down, AK‐7 barely increased RTN4B or decreased BACE1 (Figure 5i) in the hippocampus of *APP*/*PS1* mice, which is consistent with our in vitro results (Figure 3g). Besides, the assay for the Aβ level showed a consistent effect. When RTN4B was knocked down, the effect of reduced Aβ by AK7 administration was dampened (Figure 5j,k). The results from Elisa assay also confirmed that AK‐7 prominently reduced soluble and insoluble Aβ42 levels in the hippocampus, and the RTN4B knockdown increased Aβ42 to a level similar to that in the untreated group (Figure 5l). In summary, these in vitro and in vivo results suggested that this freshly identified SIRT2/RTN4B/BACE1 pathological pathway is one of the critical mechanisms for the improvement of SIRT2 on AD.

## 3 DISCUSSION

Here, we report that the inhibition of SIRT2 deacetylase activity has a beneficial effect in the AD mouse model, the reduction in Aβ pathology via a decrease in BACE1 protein level contributed to the improvement of cognitive behavior. As for the mechanism, deacetylation RTN4B by SIRT2 contributed to a reduction in RTN4B and therefore leading to the disturbance of BACE1. Our report suggest that SIRT2/RTN4B/BACE1 is a pathological pathway in AD, and SIRT2 is a promising therapeutic target for AD treatment.

**FIGURE 6:**
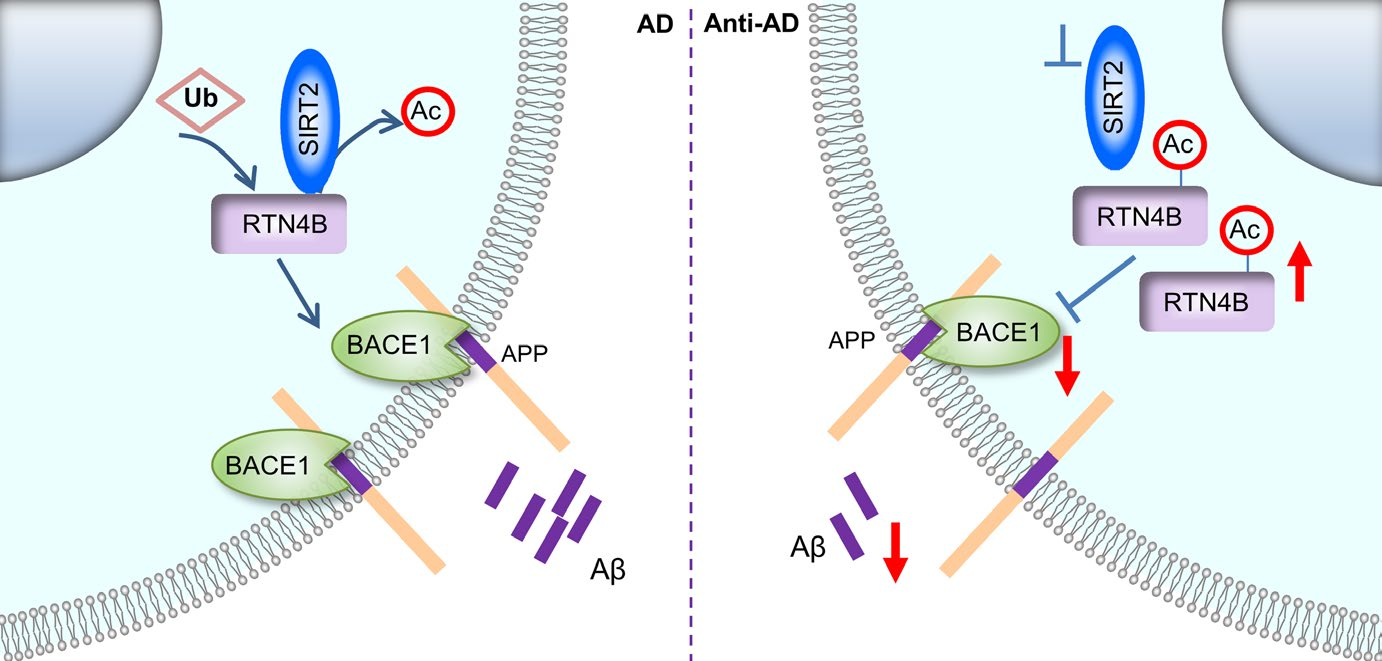
Schematic diagram depicting the possible mechanisms. In AD pathology, the imbalance of the APP metabolic pathway caused the abnormally accumulation and aggregation of Aβ in the brain, and the abnormal increase in the activity and expression of BACE1 is one of the most important causes for the Aβ accumulation in AD. Our study found that the inhibition of SIRT2 induces ubiquitination and degradation of RTN4B by deacetylating RTN4B, then the upregulation of RTN4B leading to the reduction of BACE1, suppress Aβ production, ultimately alleviates the cognitive decline of AD.

To evaluate the role of SIRT2 in cognitive impairment in AD, we first considered that reducing SIRT2 activity rather than its expression levels might represent a more promising therapeutic target for AD. Although AD is a neurodegenerative disease, non‐invasive treatment with blood‐brain barrier permeable drugs is still the optimal choice. Our results were consistent with previous reports that AK‐7 administration can improve cognition in two other AD mice models (Biella et al., 2016) and that another SIRT2 inhibitor can improve cognition in a senescence‐accelerated mouse model (Diaz‐Perdigon et al., 2020). Furthermore, we confirmed that SIRT2 inhibition reduces the Aβ burden, mainly by curtailing APP processing, via the regulation of BACE1 in vivo, which was also supported by another study that showed SIRT2 inhibition decreased Aβ levels in vitro (Biella et al., 2016). Considering SIRT2 and SIRT1 have been reported showed an opposite effect on neurodegeneration (Donmez & Outeiro, 2013), for eliminating the possible feedback increase of SIRT1 caused by SIRT2 inhibition, we also assayed the expression level of SIRT1 in the context of SIRT2 pharmacological or genetic inhibition. The result showed that the SIRT2 repress does not significantly disturb SIRT1 (Appendix S1, Figure S11). Recently, the setback in the development of drugs that target Aβ and BACE1 challenged the classical Aβ hypothesis of AD. However, the resurrection of Aducanumab (Biogen), which targets Aβ, combining with the exclusive genetic evidence, has reignited the hope for Aβ‐targeted therapy (Schneider, 2020; Selkoe, 2019). In this study, we found that SIRT2 regulated both Aβ40 and Aβ42 production (Figure 5l, Appendix S1, Figures S12 and S11), and rescued the death of neuron in the hippocampus of AD mouse model (Appendix S1, Figure S13). We further found that SIRT2 influenced Aβ production by regulated BACE1, which is one of the compelling new pathological pathways for AD. BACE1 has acetylation sites, which may be deacetylated (Ko & Puglielli, 2009), so we tried to verify whether SIRT2 has direct interactions with BACE1. However, our Co‐IP‐MS database did not identify the BACE1 protein. Nevertheless, we did further identify the candidate protein RTN4B through the informatics analysis, which may link the SIRT2 on BACE1 regulation (Figure 6).

The interaction between SIRT2 and RTN4B was firstly confirmed in our study. Our MS results identified 285 certain proteins that may be candidate targets of SIRT2 in neuronal cell lines (Appendix S1, Table S1). Then, we further locked five candidates that may serve as SIRT2 targets in AD progression by informatics analysis and finally identified RTN4 as the most impressive candidate for that it may interact with BACE1 (He et al., 2004; Murayama et al., 2006). Besides, a previous study that using metabolic labeling quantitative MS also suggested that RTN4 may transiently target SIRT2 (Budayeva & Cristea, 2016), and this possible interaction was also identified in other proteomic studies (Huttlin et al., 2015), supporting our results. RTN4, also known as Nogo, is one member of the RTN family and has mainly three isoforms (RTN4A, RTN4B, RTN4C), which are abundant in the nervous system (Chen et al., 2000). Although our screening results and previous reports suggest that SIRT2 may interact with RTN4, it is unclear which subtype of RTN4 is involved in this interaction. We found that SIRT2 strongly interacts with RTN4B and slightly interacts with RTN4A, but we did not observe the interaction between SIRT2 and RTN4C in our Co‐IP assay (Appendix S1, Figure S7). So, we focused on RTN4B and further confirmed that RTN4B interacts with SIRT2.

Concerning BACE1 and RTN4B, several studies suggested that RTN family members are the binding partners of BACE1 (Murayama et al., 2006). We provide further evidence that RTN4B could interact with BACE1, and further influence its protein level, and contribute to Aβ production. It is worth noting that BACE1 protein levels, but not mRNA levels, remarkably increased in AD pathology (Holsinger, McLean, Beyreuther, Masters, & Evin, 2002; Yang et al., 2003), and BACE1 is also predominantly present in and plays roles in the endoplasmic reticulum, plasma membrane, and endosomes (Huse, Pijak, Leslie, Lee, & Doms, 2000). It is consistent with the subcellular localization of RTN4B, which supports the conclusion that RTN4B interacts with and disturbs BACE1 in the cytoplasm. RTN3 negatively regulates BACE1 abundance in vitro and reduces the localization of BACE1 in axons (Deng et al., 2013). As the author speculated, BACE1 reduction in axons led to more BACE1 retention in some where resident ubiquitin‐lysosome can degrade it (Shi et al., 2014). Considering RTN4B not only share the same C‐terminal domain with RTN3 which interacts with BACE1 but also has a similar feature with RTN3 as an endoplasmic reticulum protein, we speculate that RTN4B may regulate BACE1 negatively by the same pattern with RTN3. SIRT2 interacts with and deacetylates RTN4B which, in turn, increases the ubiquitination and degradation.

Some limitations of this study should be addressed. In this study, we reduced RTN4B protein levels in vivo by knocking down RTN4B using shRNA, and when RTN4B was decreased, the effect of SIRT2 inhibition on AD progression was not abrogated completely, merely dampened. Two possible reasons may account for this result. First, it may be caused by the incomplete inhibition of RTN4B. Second, many substrates of SIRT2 have been identified; SIRT2 inhibition may also occur in other pathways at the same time. For example, the other four candidate proteins, from our proteomic screen, are all mitochondrial proteins, and several reports suggested that SIRT2 could influence neurodegeneration via mitochondrial function or autophagy (Liu et al., 2017). Thus, we cannot rule out the possibility that other pathways were impacted by SIRT2 inhibition in AD. Third, we did not determine the interaction between SIRT2 and RTN4B in vivo and identified the certain acetylation sites of RTN4B in this report, and this part will be clarified in future research to strengthen the conclusion.

In conclusion, data from our study indicate that inhibition of SIRT2 deacetylase activity could be an attractive target to mitigate neurodegeneration in AD. SIRT2 regulates BACE1 by deacetylating RTN4B, which, in turn, influences Aβ production and aggregation, ultimately alleviates the cognitive decline of AD. Considering the abundance of SIRT2 in the brain and it mainly plays a regulatory role in physiological processes, (Wang, Yang, Hong, Chen, & Cui, 2019) strongly suggest that it may represent a promising target for the development of new treatments for AD. In the future, a deeper understanding of the role SIRT2 plays in AD will be needed to provide confidence in developing it as a therapeutic target for AD.

## 4 EXPERIMENTAL PROCEDURES

### 4.1 Plasmids, viruses, chemicals, and antibodies

The plasmids encoding Flag‐SIRT2, HA‐SIRT2, HA‐SIRT2H187Y, Flag‐RTN4A, Flag‐RTN4B, Flag‐RTN4C, and HA‐BACE1 were constructed by Longqian Biotech (China). HA‐Ub was purchased from Addgene (USA). AAV2/9‐hSyn‐Rtn4b‐3×flag and AAV2/9‐Rtn4b shRNA were constructed and packaged by Hanbio Biotechnology Co., Ltd. LV‐SIRT2‐shRNA1 and LV‐SIRT2‐shRNA2 were constructed and packaged by Cyagen Biosciences (China). CHX was purchased from Abcam (UK). AGK2 was purchased from Sigma (USA), and AK‐7 was purchased from MCE (USA). TSA was purchased from MCE (USA). The antibodies employed in this study are listed in Appendix S1, Table S2.

### 4.2 Cell culture, transfection, and drug treatment

The human embryonic kidney 293T (HEK293T), human neuroblastoma SY5Y, human neuroglioma H4, and mouse hippocampal neuron HT22 cell lines were used in this study, and details of culture and treatments methods were described in Appendix S1.

### 4.3 Animals

Male *APP*/*PS1* double‐transgenic mouse lines, which harbor human *APP*swe (Swedish mutations K595N/M596L) and *PS1* with an exon 9 deletion (PS1dE9) under the control of the mouse prion promoter, were purchased from the Model Animal Research Center of Nanjing University. *Sirt2 *knockout (*Sirt2* ^−/−^) mice were generated using a gene‐trapping method with a C57BL/6 background and purchased from Cyagen Biosciences. All mice had free access to food and water and were housed in a pathogen‐free environment in a reversed day/night cycle. Bodyweight, food and water intake, and overall general health were assessed every week. 7‐month‐old *APP*/*PS1* and age‐matched C57BL/6 mice were used in this study. 15‐month‐old *Sirt2 *knockout mice and WT mice were used. *APP*/*PS1* and C57BL/6 mice were injected intraperitoneally with 10 mg/kg AK‐7, which was confirmed to specifically inhibit the activity of SIRT2 and effectively penetrate the blood‐brain barrier to slow the disease progression in mouse models of neurodegeneration or vehicle (10% DMSO, 90% saline) twice a day for 3 weeks.

### 4.4 Brain tissue preparation

Mice were deeply anesthetized with chloral hydrate and transcardially perfused with saline. Brains were removed rapidly and dissected into two hemispheres. One hemisphere was embedded in OCT compound immediately before freezing and cutting and stored at −80°C. The other hemisphere was immediately submerged in ice‐cold phosphate‐buffered saline (PBS), and the cortex and hippocampus were carefully dissected. The dissected tissues were snap‐frozen in liquid nitrogen and stored at −80°C until use in the biochemical analyses.

### 4.5 Immunoprecipitation and western blot

Cells or tissues were lysed with lysis buffer containing protease inhibitor cocktail (Sigma) and phenylmethylsulfonyl fluoride (PMSF) on ice and then subjected to a BCA assay (Pierce). For IP, whole‐cell lysates were incubated with specific antibodies overnight at 4 ℃, followed by incubation with protein A/G agarose beads (GE) for 3 hr at 4°C and washing with lysis buffer. Thereafter, the beads were boiled with 2× sample buffer at 95°C for 3 min before western blot. For western blot, details were described in Appendix S1.

### 4.6 ELISA assay

Aβ from *APP*/*PS* mouse brain was measured by ELISA. See Appendix S1 for details.

### 4.7 Real‐time quantitative PCR

Total RNA was extracted using Trizol reagent (Invitrogen), and 800 ng RNA was used as a template to convert to complementary DNA with the PrimeScriptTM RT reagent kit (Takara). Quantitative real‐time PCR was performed using SYBR Green by LightCycler96 (Roche). Relative abundance was calculated using the ΔΔCt method normalized to the housekeeping gene. The primers are listed in Appendix S1, Table S3.

### 4.8 Stereotaxic injection of the virus

Mice were anesthetized with 5% chloral hydrate (8 μl/g). Bilateral intracerebral injection of AAV Vector was performed stereotactically using the following coordinates: anterior‐posterior, −2.3 mm; medial‐lateral, ±1.8 mm; and dorsal‐ventral, −2.2 mm relative to bregma. A volume of 1 μl of virus (virus titer: 10^12^ vg/ml) was injected into each point at a rate of 0.1 μl/min using 1 μl syringes with a fixed needle. The needle was kept in place for 5 min before it was removed slowly. The mice were placed on a heating pad until they began to recover from the surgery. All animal experiments were approved by the Animal Care and Use Committee of Guangdong Medical University and performed in compliance with the National Institutes of Health Guide for the Care and Use of Laboratory Animals.

### 4.9 Morris water maze test (MWM)

Spatial learning and memory abilities were evaluated with the MWM with some modifications, details were described in Appendix S1.

### 4.10 Novel object recognition

The NOR test is based on the innate tendency of rodents to explore a novel object more often than a familiar one, details were described in Appendix S1. The recognition index = time spent exploring the novel object/ the time spent exploring both objects. The discrimination index = time (spent on the novel object‐ time spent on the old object)/ the time spent exploring both objects.

### 4.11 Immunofluorescence and laser scanning confocal microscopy

Coronal sections were prepared from fresh‐frozen sections of mouse brain hemispheres. Sections (10 μm) were fixed with acetone/methanol for 10 min at room temperature and blocked with 0.1% Triton X‐100/10% goat serum for 30 min at room temperature. Sections were then incubated with primary antibody at 4 °C overnight and stained with fluorescently conjugated secondary antibodies at room temperature for 1 hr. A final counterstaining with DAPI was performed for 3 min. All sections were washed with PBS, and images were acquired using a confocal microscope (FV3000, Olympus).

### 4.12 Detection of apoptosis by TdT‐mediated dUTP nick‐end labeling (TUNEL) assay

Coronal sections were prepared from fresh‐frozen sections of mouse brain hemispheres. Sections (10 μm) were fixed with 4% paraformaldehyde for 45 min at room temperature and then measured using kits (Beyotime) according to the manufacturer's instructions. Images were acquired using a confocal microscope (FV3000, Olympus).

### 4.13 Mass spectrometry analysis of protein mixtures

Whole‐cell lysates of SY5Y cells transfected with Flag‐SIRT2 or vector were IPed by Flag‐conjugated beads (Sigma) and eluted using a 3× Flag peptide. The immunoprecipitate was digested overnight at 37°C with trypsin and then lyophilized to dryness. After separation using a C18 column, the peptides were identified by MS (Thermo Scientific Q Exactive). Raw data from the MS analysis were extracted and subjected to a search against the UniProt Swiss‐Prot sequence database (HUMAN_2017.10.29_UniProt.fasta).

### 4.14 Statistical analysis

Data are presented as mean ± standard error of the mean (*SEM*). Statistical analyses were performed by Student's *t* test for the comparison of two groups; by one‐way ANOVA with Tukey's post hoc test for the comparison of three groups with one independent variable; and by two‐way ANOVA for groups with two independent variables. Statistical analysis was performed using GraphPad Prism 6 (GraphPad Software). ImageJ software was used to quantify the expression of the protein.

## CONFLICT OF INTEREST

The authors declare no conflict of interest.

## AUTHOR CONTRIBUTIONS

Y.W. participated in experiments designed, involved in cell culture, analyzed and interpreted data, prepared figures, and wrote the manuscript; J.Y. performed mice experiments and ELISA assays; T.H. assisted in the mice experiments and performed the immunostaining experiments and acquired images; Y.S. performed the western blot experiments and quantitative PCR studies; H. H. and F.C. provided helpful discussions; X.C. performed mice management; H.C. helped draw the schematic diagrams; S.D. performed the bioinformatics analysis; L.C. and T.Y. provided guidance, helped design the experiment, supervised the overall project, and edited the manuscript.

## Supporting information

Supplementary Material

## Data Availability

The authors declare that the authors provide all data included in this study upon request when there is a reasonable request.
