# Supplementary material for: RTN4B‐mediated suppression of Sirtuin 2 activity ameliorates β‐amyloid pathology and cognitive impairment in Alzheimer's disease mouse model
Source: Aging Cell. 2020 Jul 23;19(8):e13194. doi: 10.1111/acel.13194 (PMC7431833; doi:10.1111/acel.13194)
Supplement: Supplementary file 1 — Supplementary Material [file ACEL-19-e13194-s001.pdf]

Supplemental Figure 1

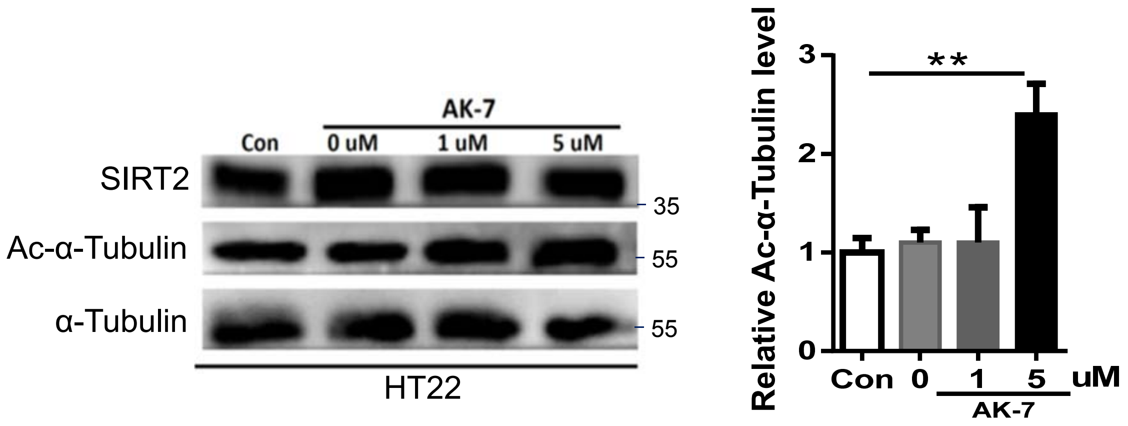

Figure S1. Representative western blot images and relative expression of Ac-α-Tubulin in HT22 cells treated with 1, 5 µM AK-7 for 12h. Data are presented SEM of 3 independent experiments. ** p<0.01. One-way ANOVA with Tukey's post hoc test.

Supplemental Figure 2

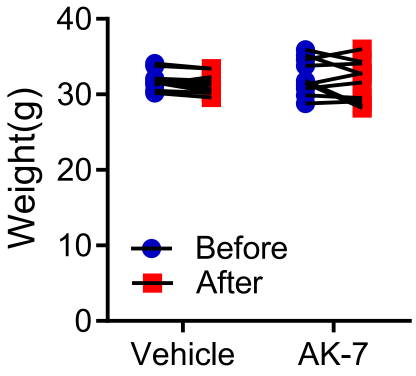

Figure S2. The bodyweight of mice before and after treated with AK-7 or Vehicle (n=9 per group). Paired Student's *t*-test.

Supplemental Figure 3

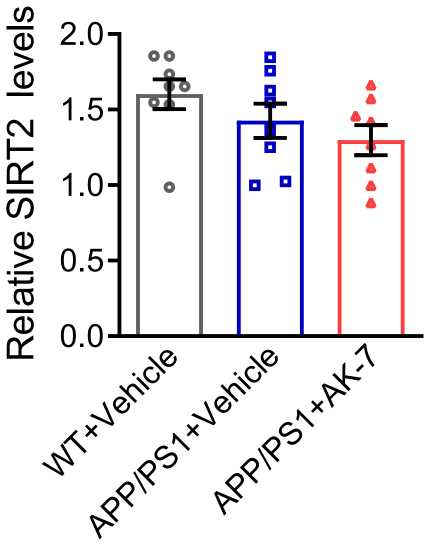

Figure S3. Relative protein expression of SIRT2 was analyzed from Figure 1j. Data are presented as SEM. One-way ANOVA with Tukey's post hoc test.

Supplemental Figure 4

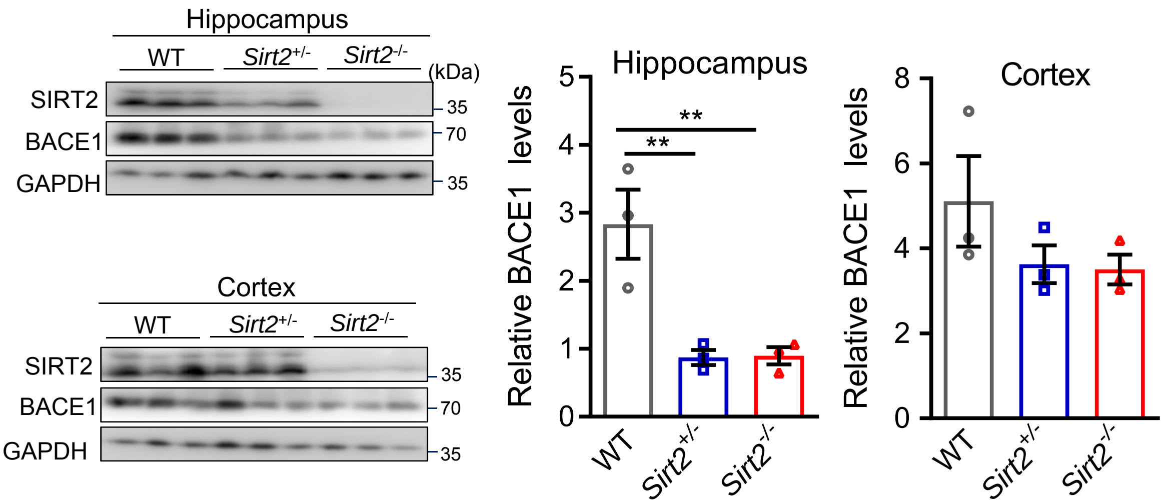

Figure S4. Representative western blot images and relative expression of SIRT2 in the hippocampus and cortex of 15-month-old WT, *Sirt2*$^{+/-}$, *Sirt2*$^{-/-}$ mice (n=3 per group). Data are presented SEM. ** p<0.01. One-way ANOVA with Tukey's post hoc test.

Supplemental Figure 5

(a)

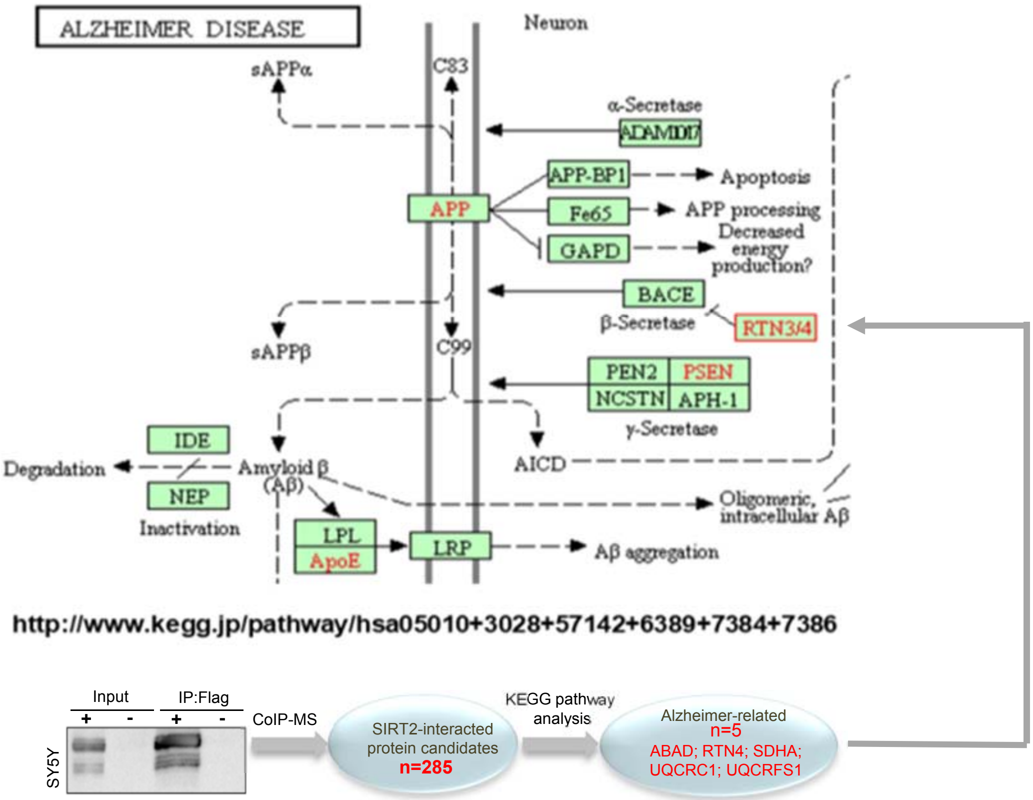

Figure S5. (a). Experimental scheme to identify target protein. (b). Gene ontology (GO) term enrichment analysis of the 285 identified proteins. (c). KEGG pathway analysis of the 285 identified proteins.

Supplemental Figure 5

(b)

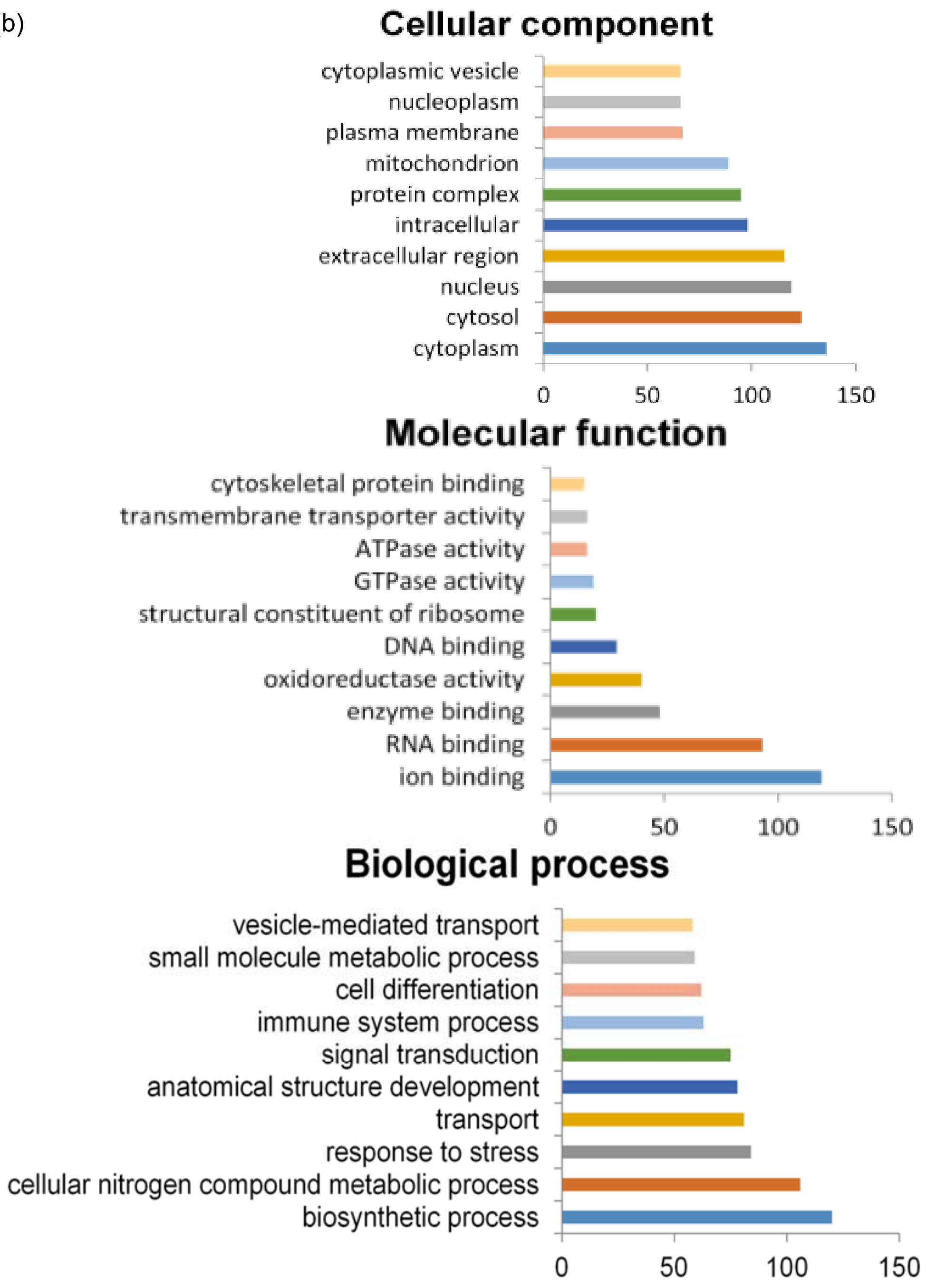

Supplemental Figure 5

(c)

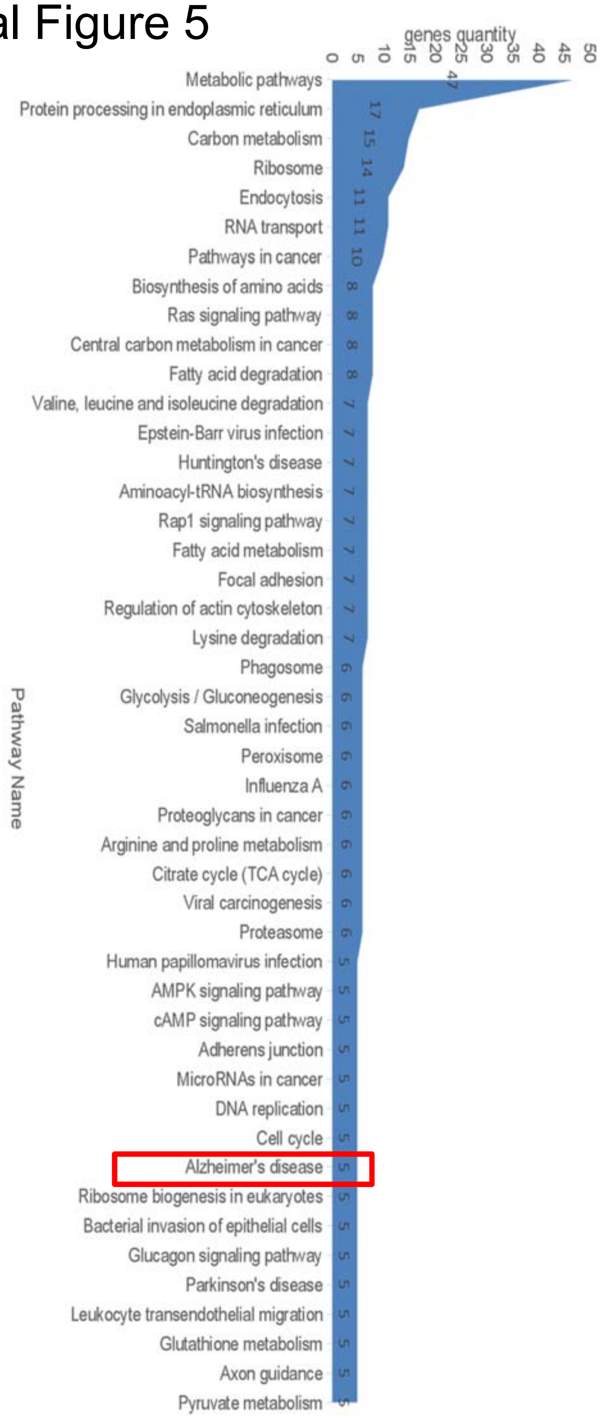

Supplemental Figure 6

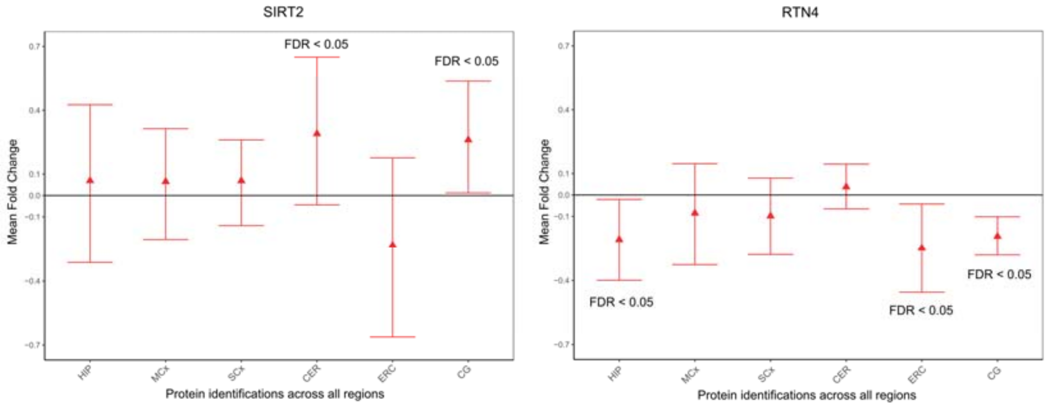

Figure S6. SIRT2 and RTN4 protein expression changes within the AD brain from six functionally distinct regions of human post-mortem brain. (a) SIRT2 protein increase in both CER (FDR = 0.045) and CG (FDR = 0.03). (b) RTN4 reduce in three brain regions, including HIP (FDR = 0.024), ERC (FDR = 0.018), and CG (FDR = 0.003) (data from PXD008806). hippocampus (HIP), entorhinal cortex (ENT), cingulate gyrus (CG), sensory cortex (SCx), motor cortex (MCx) and cerebellum (CB). (n = 9 AD cases, n = 9 asymptomatic controls)

Supplemental Figure 7

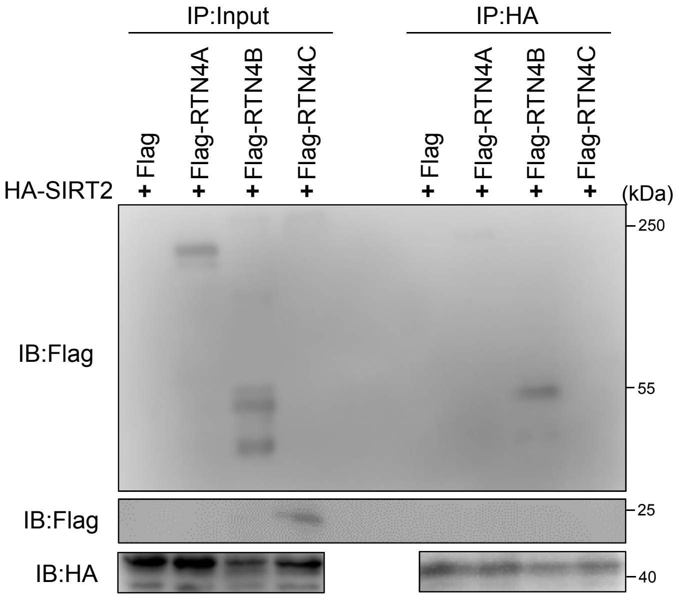

Figure S7. HA-SIRT2 and either Flag-RTN4A, or Flag-RTN4B, or Flag-RTN4C plasmids were transiently transfected into 293T cells. Total proteins were IPed with HA antibodies and subsequently by western blot with HA or Flag antibodies. Data are representative of 2~3 independent experiments.

Supplemental Figure 8

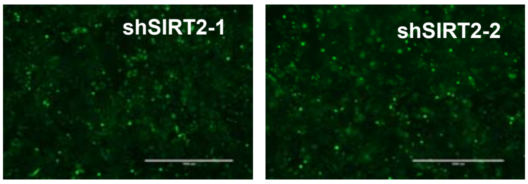

pLV[shRNA]-EGFP:T2A:Puro-U6>hSIRT2[shRNA]

SIRT2 RNAi region

sh1：TAT GACAACCTAGAGAAGTACCTCGAGGTACTTCTCTAGGTTGTCATA

sh2：AAGTAGTGACAGATGGTTGGCCTCGAGGCCAACCATCTGTCACTACTT

Figure S8. Representative immunofluorescence images of SY5Y cells transfected with lentivirus-based delivery of SIRT2-targeting short hairpin RNA (shRNA) (Scare bar: 1000μm).

Supplemental Figure 9

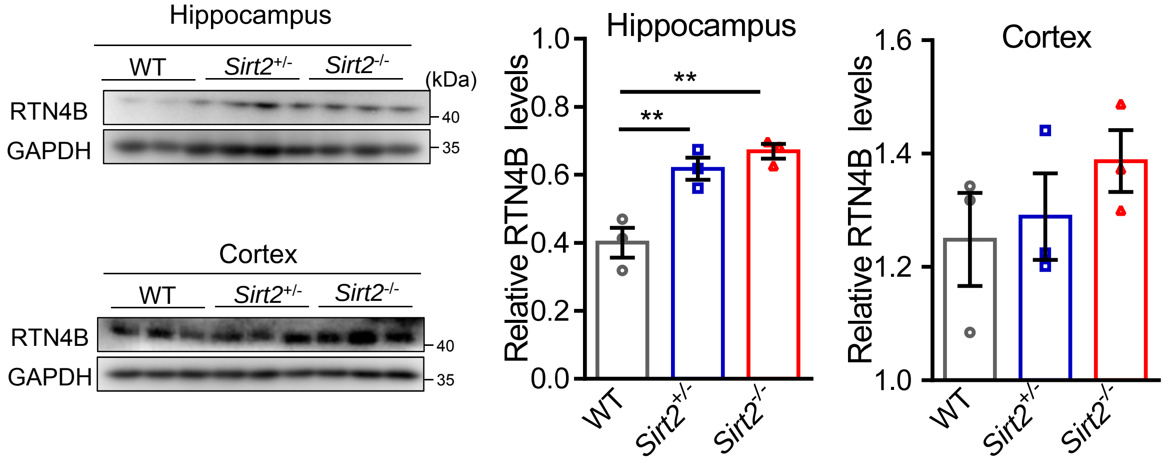

Figure S9. Representative western blot images and relative expression of RTN4B in the hippocampus and cortex of 15-month-old WT, *Sirt2*$^{+/-}$, *Sirt2*$^{-/-}$ mice (n=3 per group). Data are presented SEM. ** p<0.01. One-way ANOVA with Tukey's post hoc test.

Supplemental Figure10

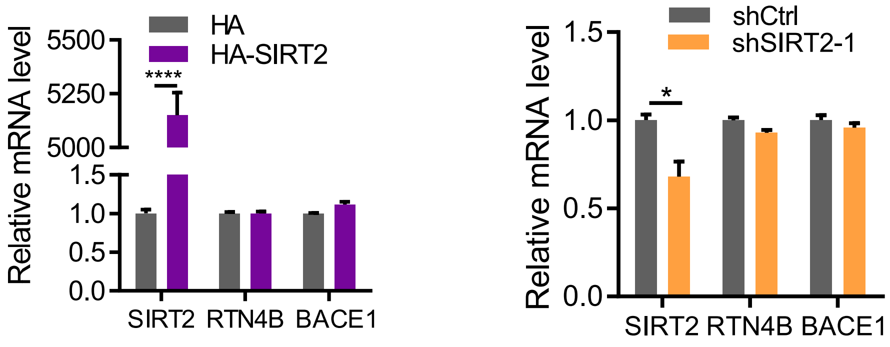

Figure S10. HA-SIRT2 or shSIRT2 were transiently transfected into SY5Y cells, and *RTN4B* and *BACE1* mRNA expression were determined by qPCR. Data are presented SEM. * p<0.05. **** p<0.0001. Non-paired Student's t-test.

Supplemental Figure11

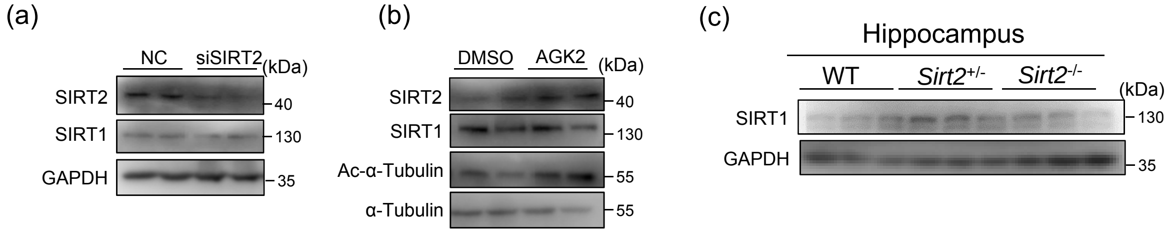

Figure S11. (a) Western blot analysis of SIRT1 expression after treatment of siSIRT2 or AGK2 in SY5Y cells. Data are representative of 2~3 independent experiments. (b) Representative western blot images and relative expression of SIRT1 in the hippocampus and cortex of 15-month-old WT, *Sirt2*$^{+/-}$, *Sirt2*$^{-/-}$ mice (n=3 per group). Data are presented SEM ** p<0.01. One-way ANOVA with Tukey's post hoc test.

Supplemental Figure12

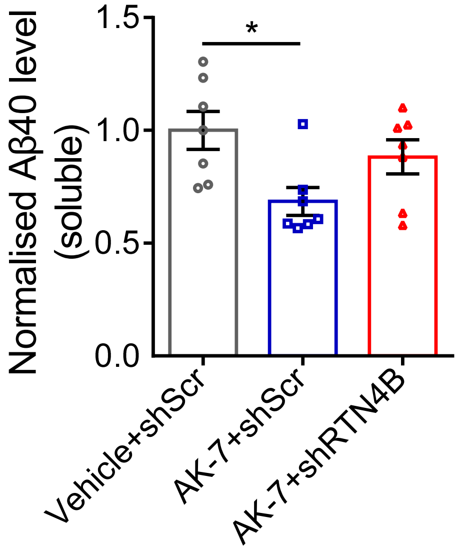

Figure S12. ELISA was used to measure soluble Aβ40 levels in the *APP/PS1* mouse brain (n=7 per group). * $p<0.05$. One-way ANOVA with Tukey's post hoc test.

Supplemental Figure13

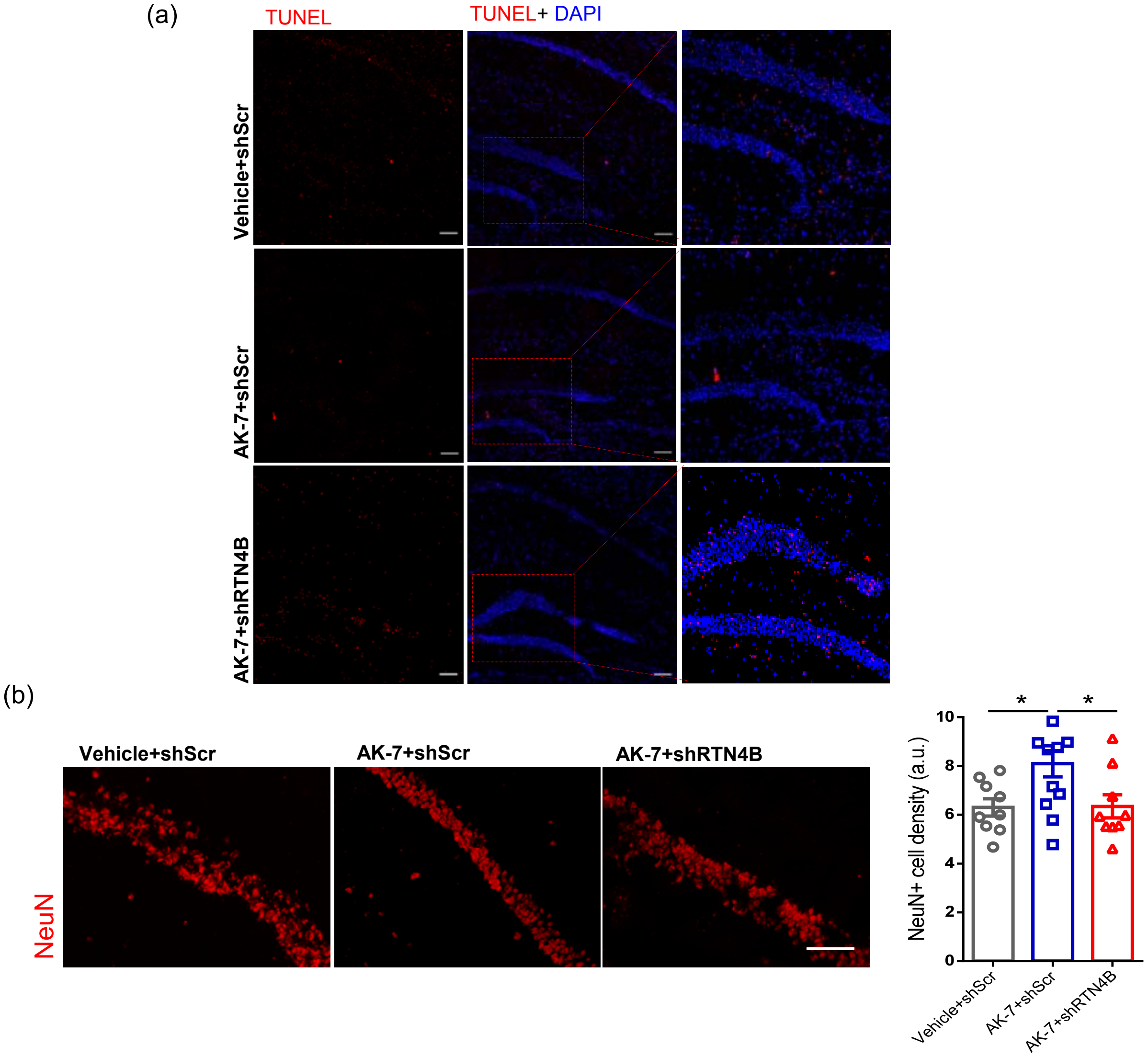

Figure S13. (a) Representative images of immunofluorescence staining of Tunel in the hippocampus of *APP/PS1* mice of indicated groups (Scale bar, 100μm). (b) Representative immunofluorescence images of NeuN staining cells from CA1 of *APP/PS1* mice of indicated groups (Scale bar, 100μm) and summary of $NeuN^+$ cell numbers (n=9~10 slices from 3 mice per group). * $p<0.05$. One-way ANOVA with Tukey's post hoc test.

Table S1. The screened SIRT2 interacting proteins (n=285) by Co-IP MS assay in SIRT2 overexpressed SH-SY5Y cell line.

**Citable Accession**	**Gene Name**	**Citable Accession**	**Gene Name**	**Citable Accession**	**Gene Name**
Q9NZM1	GN=MYOF	Q05D32	GN=CTDSPL2	P23921	GN=RRM1
Q7KZF4	GN=SND1	Q9Y4L1	GN=HYOU1	Q9Y3T9	GN=NOC2L
P16401	GN=HIST1H1B	Q8N983	GN=MRPL43	P41252	GN=IARS
Q9P015	GN=MRPL15	P57740	GN=NUP107	P39656	GN=DDOST
P40939	GN=HADHA	Q7Z2W9	GN=MRPL21	P50914	GN=RPL14
Q14697	GN=GANAB	P07954	GN=FH	Q92945	GN=KHSRP
Q13423	GN=NNT	P50416	GN=CPT1A	Q8WYP5	GN=AHCTF1
P00387	GN=CYB5R3	Q9H0U6	GN=MRPL18	P50454	GN=SERPINH1
P46459	GN=NSF	P49736	GN=MCM2	Q9NQ55	GN=PPAN
P30101	GN=PDIA3	P62861	GN=FAU	Q9BYD6	GN=MRPL1
Q15084	GN=PDIA6	P23528	GN=CFL1	Q9BYD3	GN=MRPL4
Q00610	GN=CLTC	P07737	GN=PFN1	Q9BUJ2	GN=HNRNPUL1
Q13310	GN=PABPC4	O00116	GN=AGPS	Q8WXX5	GN=DNAJC9
O43854	GN=EDIL3	Q5T653	GN=MRPL2	Q4G0N4	GN=NADK2
Q15942	GN=ZYX	P08133	GN=ANXA6	Q27J81	GN=INF2
P27824	GN=CANX	P53621	GN=COPA	P23381	GN=WARS
P00533	GN=EGFR	P55265	GN=ADAR	P08754	GN=GNAI3
P84095	GN=RHOG	P51659	GN=HSD17B4	O94973	GN=AP2A2
P24752	GN=ACAT1	P01275	GN=GCG	O43837	GN=IDH3B
P51149	GN=RAB7A	P11233	GN=RALA	O43242	GN=PSMD3
P13804	GN=ETFA	Q13405	GN=MRPL49	A0A0A0MRZ7	GN=IGKV2D-26
Q99714	GN=HSD17B10	P17812	GN=CTPS1	Q9Y2Q3	GN=GSTK1
P25205	GN=MCM3	P49257	GN=LMAN1	Q9NQ39	GN=RPS10P5
O75683	GN=SURF6	P53396	GN=ACLY	Q9BUR5	GN=APOO
P54886	GN=ALDH18A1	Q8NFH5	GN=NUP35	Q14197	GN=MRPL58
P51148	GN=RAB5C	P08559	GN=PDHA1	Q02978	GN=SLC25A11
P26640	GN=VARS	Q9BSJ8	GN=ESYT1	P80723	GN=BASP1
Q8TAE8	GN=GADD45GIP1	P49755	GN=TMED10	P51151	GN=RAB9A
P08865	GN=RPSA	Q00341	GN=HDLBP	P28066	GN=PSMA5
P61019	GN=RAB2A	P41091	GN=EIF2S3	P04181	GN=OAT
Q99832	GN=CCT7	Q96S66	GN=CLCC1	O14828	GN=SCAMP3
Q8NBX0	GN=SCCPDH	O75746	GN=SLC25A12	Q9Y4W6	GN=AFG3L2
Q9H0A0	GN=NAT10	P61224	GN=RAP1B	Q9P0L0	GN=VAPA
P27797	GN=CALR	P00505	GN=GOT2	P21796	GN=VDAC1
Q02809	GN=PLOD1	Q9H9J2	GN=MRPL44	Q8NBJ5	GN=COLGALT1
P46940	GN=IQGAP1	P78417	GN=GSTO1	Q9UBB4	GN=ATXN10
Q9HB71	GN=CACYBP	Q5SSJ5	GN=HP1BP3	Q7L2E3	GN=DHX30
O43707	GN=ACTN4	Q14204	GN=DYNC1H1	Q9H0S4	GN=DDX47
P49748	GN=ACADVL	O94925	GN=GLS	O75955	GN=FLOT1
P31040	GN=SDHA	Q9Y657	GN=SPIN1	Q8N766	GN=EMC1

Citable Accession	Gene Name	Citable Accession	Gene Name	Citable Accession	Gene Name
P61106	GN=RAB14	Q9H6F5	GN=CCDC86	Q71UM5	GN=RPS27L
Q9Y6C9	GN=MTCH2	P52292	GN=KPNA2	O14818	GN=PSMA7
P06744	GN=GPI	Q9UJS0	GN=SLC25A13	P55786	GN=NPEPPS
P62820	GN=RAB1A	P27694	GN=RPA1	P19367	GN=HK1
Q16576	GN=RBBP7	Q9NYK5	GN=MRPL39	P16435	GN=POR
Q13838	GN=DDX39B	Q9GZT3	GN=SLIRP	P12429	GN=ANXA3
P60228	GN=EIF3E	Q13011	GN=ECH1	P11166	GN=SLC2A1
P62244	GN=RPS15A	P61586	GN=RHOA	O15427	GN=SLC16A3
P31930	GN=UQCRC1	Q9NSE4	GN=IARS2	O00303	GN=EIF3F
O76021	GN=RSL1D1	Q96C36	GN=PYCR2	Q9Y262	GN=EIF3L
P26196	GN=DDX6	P50213	GN=IDH3A	Q9UNL2	GN=SSR3
Q9UNF1	GN=MAGED2	Q05682	GN=CALD1	Q9UBS4	GN=DNAJB11
Q96GQ7	GN=DDX27	Q96KR1	GN=ZFR	Q9NQT4	GN=EXOSC5
P50750	GN=CDK9	Q14244	GN=MAP7	Q9HC07	GN=TMEM165
P34897	GN=SHMT2	P55060	GN=CSE1L	Q9H7Z7	GN=PTGES2
Q96DV4	GN=MRPL38	P33992	GN=MCM5	Q9BVC6	GN=TMEM109
O60832	GN=DKC1	O60341	GN=KDM1A	Q9BU61	GN=NDUFAF3
Q01813	GN=PFKP	P56192	GN=MARS	Q99873	GN=PRMT1
P19525	GN=EIF2AK2	P35613	GN=BSG	Q8WXI9	GN=GATAD2B
Q99613	GN=EIF3C	O14732	GN=IMPA2	Q8WXE9	GN=STON2
P21291	GN=CSRP1	Q9BZG1	GN=RAB34	Q8TC12	GN=RDH11
Q9UHD8	GN=SEPT9	Q96LJ7	GN=DHRS1	Q8N5N7	GN=MRPL50
P08240	GN=SRPRA	O00469	GN=PLOD2	Q6PIU2	GN=NCEH1
Q04637	GN=EIF4G1	Q5UIP0	GN=RIF1	Q5RI15	GN=COX20
Q8WTT2	GN=NOC3L	P32004	GN=L1CAM	Q5BKZ1	GN=ZNF326
Q6YN16	GN=HSDL2	Q9Y5J1	GN=UTP18	Q14165	GN=MLEC
P38117	GN=ETFB	Q9BQC6	GN=MRPL57	P61966	GN=AP1S1
Q9HBL7	GN=PLGRKT	Q8N556	GN=AFAP1	P53618	GN=COPB1
Q99623	GN=PHB2	Q3ZCQ8	GN=TIMM50	P52209	GN=PGD
Q9NZ01	GN=TECR	P51571	GN=SSR4	P51572	GN=BCAP31
Q15717	GN=ELAVL1	O95573	GN=ACSL3	P49189	GN=ALDH9A1
P25789	GN=PSMA4	O75694	GN=NUP155	P31153	GN=MAT2A
O15479	GN=MAGEB2	Q9Y4P3	GN=TBL2	P16152	GN=CBR1
Q9UKD2	GN=MRTO4	Q9NQC3	GN=RTN4	P11310	GN=ACADM
P28288	GN=ABCD3	Q9H8H3	GN=METTL7A	O96008	GN=TOMM40
P20339	GN=RAB5A	Q9BYC9	GN=MRPL20	O60831	GN=PRAF2
Q9H3N1	GN=TMX1	Q9BY44	GN=EIF2A	O15446	GN=CD3EAP
Q8TCJ2	GN=STT3B	Q9BRX8	GN=FAM213A	O00487	GN=PSMD14
P43304	GN=GPD2	Q92973	GN=TNPO1	O00461	GN=GOLIM4
Q9P0M9	GN=MRPL27	Q8TDD1	GN=DDX54	O00186	GN=STXBP3
Q9H6R4	GN=NOL6	Q8NBJ7	GN=SUMF2	P51809	GN=VAMP7
P16070	GN=CD44	Q5T3I0	GN=GPATCH4	Q9HDC5	GN=JPH1

Citable Accession	Gene Name	Citable Accession	Gene Name	Citable Accession	Gene Name
Q6P1J9	GN=CDC73	Q15008	GN=PSMD6	Q92900	GN=UPF1
P11413	GN=G6PD	Q13393	GN=PLD1	Q9Y6B6	GN=SAR1B
O60282	GN=KIF5C	P62847	GN=RPS24	Q9H6N6	GN=MYH16
P00491	GN=PNP	P60953	GN=CDC42	Q96G23	GN=CERS2
P28370	GN=SMARCA1	P46977	GN=STT3A	Q9NSD9	GN=FARSB
P61009	GN=SPCS3	Q9UBQ5	GN=EIF3K	Q96HC4	GN=PDLIM5
Q9NPJ3	GN=ACOT13	Q5JWF2	GN=GNAS	Q15070	GN=OXA1L
Q96CS3	GN=FAF2	Q9BTU6	GN=PI4K2A	P11388	GN=TOP2A
P26639	GN=TARS	Q9H6W3	GN=RIOX1	Q9NZE8	GN=MRPL35
Q8N6T3	GN=ARFGAP1	P55809	GN=OXCT1	P51398	GN=DAP3
Q9H173	GN=SIL1	P33993	GN=MCM7	P05026	GN=ATP1B1
Q9NW13	GN=RBM28	P51648	GN=ALDH3A2	O75369	GN=FLNB
P26358	GN=DNMT1	P47985	GN=UQCRFS1	Q92769	GN=HDAC2

Table S2 Antibodies used in this study.

Antibodies	Type	Dilution	Source
FLAG	Mouse/mono	1:1000 for WB	Abbkine
FLAG	Rabbit/poly	1:100 for IP	Abbkine
HA	Mouse/mono	1:1000 for WB	Abbkine
HA	Rabbit/poly	1:100 for IP	Abbkine
GAPDH	Rabbit/ mono	1:1000 for WB	Cell Signaling Technology
SIRT2	Rabbit/poly	1:1000 for WB	Sigma
SIRT2	Mouse/mono	1:10 for IF	Santa Cruz
RTN4B	Rabbit/ mono	1:1000 for WB<br>1:200 for IF	Abcam
RTN4B	Mouse/mono	1:500 for WB<br>1:100 for IF	Santa Cruz
BACE1	Rabbit/ mono	1:1000 for WB<br>1:200 for IF	Abcam
APP	Mouse/mono	1:500 for WB	Millipore
Acetylated-Lysine	Rabbit/poly	1:1000 for WB	Cell Signaling Technology
Ac-α-tubulin	Mouse/mono	1:1000 for WB	Santa Cruz
α-Tubulin	Rabbit/poly	1:1000 for WB	Abcam
Aβ1-42	Rabbit/mono	1:1000 for WB<br>1:200 for IF	Abcam
ADAM10	Rabbit/poly	1:1000 for WB	Abcam
NeuN	Rabbit/poly	1:400 for IF	Abcam
SIRT1	Rabbit/poly	1:1000 for WB	Beyotime

Table S3. Primers sequence for qPCR.

**Gene**	**Sequence**
hSIRT2	Forward: CACGCAGAACATAGATACCCTG
	Reverse: CAGTGTGATGTGTAGAAGGTGC
hRTN4B	Forward: GCAGTGTTGATGTGGGTATTT
	Reverse: CTGTGCCTGATGCCGTTC
hBACE1	Forward: ACCAACCTTCGTTTGCCCAA
	Reverse: TCTCCTAGCCAGAAACCATCAG
hGAPDH	Forward: GCACCGTCAAGGCTGAGAAC
	Reverse: TGGTGAAGACGCCAGTGGA

Table S4. siRNA sequence used in this study.

**Gene**	**siRNA Sequence**
hSIRT2	1: TATGACAACCTAGAGAAGTAC
	2: AAGTAGTGACAGATGGTTGGC
hRTN4B	GCAGTGTTGATGTGGGTATTT
mRTN4B	ACTATCAGCTTTAGGATATAT

Supplementary Experimental Procedures

Morris water maze test

The test was performed in a circular water tank (120 cm in diameter) with a platform (10 cm in diameter) placed in the center of each quadrant of the pool. Trajectories of all animals were monitored and acquired using a computerized tracking system. The procedure comprised a 1-day visible platform test, 5 days training trials, and a probe trial on the 6th day. In the visible-platform test, mice were tested for four continuous trials. In the training trials, the hidden platform was kept in one quadrant and submerged 1.5 cm below the water surface. During each trial, the mice were placed in water at one of the four starting positions and given a maximum of 90 s to escape onto the hidden platform. If a mouse could not locate the platform within 90 s, they were manually guided to it and allowed to rest there for 15 s. The time needed for an individual mouse to reach the hidden platform was recorded as the escape latency. In the probe trial, the platform was removed, and mice were given 90 s to find the original location of the platform. The time and distance spent in the target quadrant were recorded.

Novel object recognition

For the first day, mice were habituated to the empty open field (50 cm × 50 cm × 30 cm) for 10 min. During the familiarization session, mice were allowed to freely explore two identical objects for 10 min. In the test session on the following day, mice were placed back into the same box containing one familiar object and one novel object for 8 min. Trials were videotaped using an overhead camera, and the time spent exploring the object (nose being in the zone at a distance of less than 2 cm from the object) was recorded. The recognition index refers to the time spent exploring the novel object relative to the time spent exploring both objects. The discrimination index refers to the time spent exploring the novel object minus the time spent on the old object relative to the time spent exploring both objects.

western blot

For western blot, after resolving by sodium dodecyl sulfate-polyacrylamide gel electrophoresis (SDS-PAGE), proteins were transferred to a polyvinylidene fluoride membrane (Millipore). The membrane was then blocked and incubated with a specific primary antibody and HRP-coupled secondary antibody. In the detection of acetylation, the antibody that detects general

acetylated lysine residues was used first, and then the corresponding protein was detected after the membrane was stripped.

Cell culture, transfection, and drug treatment

Cells were cultured in Dulbecco’s Modified Eagle’s Medium (DMEM) supplemented with 10% fetal bovine serum (FBS) and 1% penicillin/streptomycin. The cells were cultured at 37 °C in a 5 % CO2 incubator. Cells were seeded in a 6-well culture plate overnight and co-transfected with a mixture containing a total of 2 μg of plasmids and 4 μL of Lipofectamine 2000 (Invitrogen, USA), according to the manufacturer’s protocols.

ELISA assay

Mouse hemispheres were homogenized in RIPA buffer supplemented with protease inhibitors cocktail (Sigma) and PMSF and then centrifuged. The supernatants were defined as the soluble section. The pellets were re-suspended with 70% formic acid, and the supernatants were defined as the insoluble one. Aβ from both parts were measured using available ELISA kits (IBL) according to the manufacturer’s instructions.
